# Brain network properties in chronic pain—a systematic review and meta-analysis of graph-based connectivity metrics

**DOI:** 10.3389/fnins.2025.1672542

**Published:** 2025-11-18

**Authors:** Lionel Butry, Johanna Thomä, Sigrid Elsenbruch, Adriane Icenhour, Robert Rehmann, Elena Enax-Krumova, Lara Schlaffke

**Affiliations:** 1 Department of Neurology, BG University Hospital Bergmannsheil, Ruhr University Bochum, Bochum, Germany; 2 Department of Medical Psychology and Medical Sociology, Medical Faculty, Ruhr University Bochum, Bochum, Germany; 3 Department of Neurology, Center for Translational Neuro- and Behavioral Sciences, University Hospital Essen, University of Duisburg-Essen, Essen, Germany; 4 Department of Affective Neuroscience, Medical Faculty, Ruhr University Bochum, Bochum, Germany; 5 BG University Hospital Bergmannsheil, Heimer Institute for Muscle Research, Bochum, Germany; 6 Department of Medical Engineering, FH Dortmund, University of Applied Sciences and Arts, Dortmund, Germany; 7 Ruhr University Bochum, University Hospital of Pediatrics and Adolescent Medicine, Bochum, Germany

**Keywords:** functional connectivity, structural connectivity, graph theory, brain topology, network analysis, chronic pain

## Abstract

**Introduction:**

Identifying brain topology alterations in chronic pain is a crucial step in understanding its pathophysiology. The primary objective of this systematic review and meta-analysis was to assess alterations in resting-state functional and structural global network properties in patients with chronic pain.

**Methods:**

Following the preregistration (PROSPERO CRD42024542390), databases were searched for studies comparing connectivity-based whole-brain global network properties between patients with chronic pain and healthy controls. Risk of bias was assessed using an adapted Newcastle-Ottawa scale. Random-effect meta-analyses were conducted for each global network property separately.

**Results:**

A total of 32 functional topology studies and 17 structural topology studies were included in the qualitative review, with 27 functional topology studies and 17 structural topology studies eligible for meta-analysis across nine unique structural and functional global network properties. The number of participants per meta-analysis ranged from 178 to 1,592. There was low-certainty evidence that chronic pain patients showed impairments in local efficiency of resting-state functional whole-brain topology (SMD: −0.50, 95%-CI: −0.81 to −0.19, 95%-PI: −1.38 to 0.38), and low to very low-certainty evidence that structural whole-brain topology was not altered in chronic pain across nine global network properties. The heterogeneity was high in the majority of functional (I^2^: 1–76%) and structural (I^2^: 68–97%) topology studies. Most functional (50%) and structural (65%) topology studies showed some concern regarding the risk of bias.

**Discussion:**

The meta-analyses indicate that functional but not structural whole-brain topological reorganisation is involved in the pathophysiology of chronic pain.

## 1 Introduction

Chronic pain imposes a significant personal and economic burden, affecting over 30% of the global population (Cohen et al., 2021). Unlike acute pain, which serves an adaptive function, chronic pain represents not just a prolonged form of pain but is associated with altered structural and functional neural plasticity across widespread brain regions (Kuner and Flor, 2016; Brandl et al., 2022). However, the role of these alterations is not fully understood. Investigating the underlying neuropathophysiology of chronic pain is crucial to facilitate innovations in the management of chronic pain disorders.

Advances in neuroimaging techniques and the growing adoption of network neuroscience have led to an increased focus on brain network topology in chronic pain research. Brain network topology refers to both the functional and structural organisation of the brain. The topology of a network is decisive for its function, as the principles of its information transfer capability naturally arise from its organisation (Moon et al., 2015). This is reflected in different brain topological alterations across neurological and psychological disorders such as depression, Parkinson’s disease, epilepsy, and traumatic brain injury (Kambeitz et al., 2016; Farahani et al., 2019; Xu et al., 2021; Slinger et al., 2022; Zuo et al., 2023). This raises the question whether brain topology can exhibit alterations characteristic of chronic pain.

Graph theory provides a robust mathematical framework for quantifying brain network topology (Farahani et al., 2019). In this approach, the brain network is modelled as a graph, composed of nodes connected by edges. Nodes represent predefined brain regions, and edges reflect the physical connection (structural connectivity) or synchronous neural activity (functional connectivity) between nodes (Bullmore and Sporns, 2009). Edges are defined by connectivity strength measures such as streamline count or fractional anisotropy (FA) in structural connectomes, or correlation coefficients in functional connectomes (Bullmore and Sporns, 2009). Graph theoretical metrics can be computed on a global network level, quantifying the topology of the entire brain network into a single value regarding its properties, such as integration, segregation, centrality, resilience, or small-worldness (Rubinov and Sporns, 2010). For a brief description of common global network properties, please refer to Table 1.

**Table 1:** Description of common global network properties.

Global network properties	Definition	Interpretation
Measures of integration		
Characteristic path length (L)	Average shortest path length between all pairs of nodes in a network	Lower values indicate greater overall connectedness (integration)
Global efficiency (E_glob_)	Average of the inverse shortest path length between all pairs of nodes	Higher values indicate greater integration
Measures of segregation		
Clustering coefficient (CC)	Fraction of triangles that actually exist over all possible triangles in the neighbourhood of a node (*)	Higher values indicate greater local connectedness
Modularity (Q)	Degree to which a network can be partitioned into densely connected modules	Higher values indicate stronger modular structure (segregation)
Local efficiency (E_loc_)	Efficiency of information transfer within a node’s neighbourhood (similar to E_glob_ but for nodes) (*)	Higher values indicate greater efficiency in local communities
Measures of centrality		
Average degree (Deg)	Number of edges connected to a node (*)	Higher values indicate a denser network
Other measures		
Small-worldness (SW)	Ratio comparing CC and L to a random network	Higher values indicate a simultaneously highly segregated and integrated network
Normalised mutual information (NMI)	Similarity between network partitions	Higher values indicate greater similarity

*Average across all nodes of a network.

Previous qualitative aggregation efforts reported alterations in global brain network topology in chronic pain patients compared to healthy individuals (Lenoir et al., 2021; Xin et al., 2024). Still, the certainty of evidence remains unknown as a plethora of new studies have been published, and no quantitative synthesis has been conducted so far.

The primary objective of this study was to assess differences in functional and structural connectivity-based global network properties between chronic pain patients and healthy controls. Secondarily, we examined whether these alterations varied by chronic pain type, distinguishing between chronic primary pain and chronic secondary pain. The ICD-11 defines chronic primary pain as a disease in its own right, whereas chronic secondary pain arises as a symptom of an underlying disease (Treede et al., 2019). Additionally, the influence of psychological comorbidities was explored. By synthesising existing evidence, our findings refine the current understanding of brain network topology in chronic pain.

## 2 Methods

The review was preregistered with PROSPERO (CRD42024542390), and the reporting used the PRISMA 2020 checklist.

### 2.1 Search strategy

An electronic database search of MEDLINE via PubMed, Web of Science, PsychInfo via EBSCO, CINAHL via EBSCO and Scopus was conducted. The search string had three domains (chronic pain, brain connectivity, graph analysis) combined by ‘AND’, containing MeSH categories and simple search terms (Appendix 1). The ‘Polyglot Search Translater’ was utilised to translate the search strings across databases (Clark et al., 2020). The initial search was completed from inception to April 29, 2024 and was updated on February 20, 2025. Additionally, articles which have been included in two *a priori* identified relevant systematic reviews (Lenoir et al., 2021; Xin et al., 2024) were added to the screening. The ‘Deduplicator’ software was used to remove article duplicates before screening (Forbes et al., 2024).

### 2.2 Eligibility criteria

Inclusion criteria followed the population, interventions, comparators, outcomes and study design (PICOS) framework.

Population. The population group of interest was adults (>18 years) with chronic pain, including chronic primary pain (i.e., chronic widespread, complex regional pain syndrome, headache or orofacial, visceral, musculoskeletal) and chronic secondary pain (i.e., cancer-related, postsurgical or posttraumatic, musculoskeletal, visceral, neuropathic, headache or orofacial) diagnoses. Chronic pain was defined as pain that persists or recurs for more than 3 months, following the ICD-11 (Treede et al., 2019). Pain duration of >3 months had to be reported in the sample eligibility criteria, sample characteristics or the diagnosis criteria. Articles investigating diseases associated with chronic secondary pain had to report the existence of pain in their population, either defined in the participants’ inclusion criteria or reported as a pain-specific measure.

Intervention. Studies had to assess resting-state functional connectivity of grey matter or structural connectivity of white matter using neuroimaging techniques such as resting-state functional MRI (rs-fMRI), rs-EEG, rs-MEG or diffusion-weighted MRI (dMRI). Functional connectivity had to be computed as a form of correlation between neural activity measures of brain regions at rest, irrespective of neuroimaging technique. Structural connectivity had to be computed based on diffusion MRI fibre tractography, irrespective of the diffusion tensor model used.

Comparator. The chronic pain group had to be compared with a healthy control group without chronic pain.

Outcome. Included studies had to examine graph-theoretical global network metrics of whole-brain functional or structural connectivity. Structural covariance networks based on T1-weighted MRI data and articles solely reporting region-of-interest or subnetwork analysis were excluded.

Study design. Any study with a comparative design of chronic pain and healthy controls that was published in a peer-reviewed journal in English or German was included. Longitudinal studies were included if baseline comparison was reported.

The synthesis was conducted separately for functional and structural topology.

### 2.3 Screening

Each record was assessed by two independent reviewers (LB and one of AI, LS, RR or SE) using the Systematic Review Facility (SyRF; Bahor et al., 2021). First, the title/abstract was screened, then the full text was screened to assess eligibility. Disagreements were resolved by EEK and by discussion between the assessors in the title/abstract phase and full text phase, respectively.

### 2.4 Data extraction

Data extraction was performed independently by two reviewers (LB, JT). The following information was extracted from the full texts of eligible articles: Relevant publication information (author, title, year), study demographics (sample size, sex ratio, age, pain duration, pain intensity), pain diagnosis, type of chronic pain (primary, secondary, both), assessment of psychological comorbidities, neuroimaging information (imaging modality, MRI field strength, voxel size, number of channels, frequency bandwidth, number of diffusion-gradient directions, b-value, tractography type), connectivity matrix information (type of connectivity, parcellation scheme, type and metric of edges, network thresholding, software used for global network property computation), main results (mean and standard deviation of connectivity-based global network metrics or *p*-value). Conflicts were resolved by discussion. In longitudinal studies, only baseline data were extracted. If studies reported multiple values of an outcome across different network thresholding levels or dynamic functional connectivity states, the data were pooled. In cases where data was reported as median and interquartile range, the mean and standard deviation were estimated using a widely used formula (Appendix 2; Wan et al., 2014). If outcome measures were reported solely in figures, WebPlotDigitizer v5.2 was utilised to extract the data. Although a standardised extraction protocol was employed to minimise plot digitalisation errors (Appendix 2), small variability in the digitalised plot data was expected. Therefore, this data was pooled across the two independent extractors. If outcome data could not be extracted, the corresponding author was contacted two times within 4 weeks.

### 2.5 Risk of bias and certainty in evidence assessment

There is no gold standard in assessing risk of bias in cross-sectional studies (Kelly et al., 2024). This review utilised an adapted Newcastle-Ottawa Scale for case–control studies (Stang, 2010), which has been employed in previous systematic reviews of global network properties (Lenoir et al., 2021; Slinger et al., 2022; Gao et al., 2023). It assesses the following domains: participant selection (max. 4 points), group comparability (max. 2 points) and outcome (max. 3 points; Appendix 2). A study can score between 0 and 9 points, with a higher score indicating a lower risk of bias. The points were converted to percentages and grouped into low risk of bias (71.6–100%), some concerns (28.6–71.5%) and high risk of bias (0–28.5%). The grouping thresholds ensure that only studies with relatively minor or major methodological issues are considered low or high risk of bias, respectively. Each included study was assessed by two independent assessors (LB, JT), and conflicts were resolved by discussion.

The certainty in evidence was assessed using the GRADE approach (very low-certainty to high-certainty). Risk of bias, inconsistency, imprecision and publication bias were considered to determine the certainty in each meta-analysis. A detailed description of the rating is presented in Appendix 2.

### 2.6 Statistical analysis

All analyses were performed in R v4.4.1 with the meta v8.0–2 R package (Balduzzi et al., 2019). Each global network property with at least three included studies was analysed separately for structural and functional brain topology, comparing the chronic pain group with the healthy control group. Subgroup analysis was conducted by segregating the chronic pain group into chronic primary pain and chronic secondary pain to explore sources of heterogeneity. In functional topology meta-analyses, only studies examining low-frequency fluctuations (≤ 0.1 Hz) were included to ensure comparability among the studies. To account for expected heterogeneity in study characteristics, random-effects meta-analyses with a restricted maximum-likelihood approach were performed using sample-bias corrected standardised mean difference (SMD; Hedges’g) and standard error (Viechtbauer, 2005). The 95% confidence intervals (CIs) for the random effects estimates were computed using the Hartung-Knapp method with adhoc variance correction (IQWiG6; Knapp and Hartung, 2003; IQWiG, 2022). The Hartung-Knapp-Partlett-Riley method with *ad hoc* variance correction was applied to estimate the 95% prediction intervals (PIs) if at least four studies were included in the meta-analysis (Partlett and Riley, 2017). Hedge’s g was calculated using the R package esc v0.5.1 (Lüdecke, 2019), based on the mean, standard deviation (SD) and sample size. When SDs or other measures of uncertainty were unavailable, and the authors were non-responsive, we imputed the SD by using the pooled coefficient of variation from other studies on the same outcome and converting it back to an SD. For studies that only reported *p*-values, Hedge’s g was estimated from the t- or F-statistic. In cases where non-significant p-values were reported without sufficient information to determine the direction of the effect, Hedge’s g was set to zero (Van Aert et al., 2016). This approach avoids making arbitrary assumptions about the effect size direction while ensuring that such studies contribute to the overall analysis through their standard error. When a study compared healthy controls to two or more chronic pain groups, the data were pooled to avoid sample overlap (Higgins et al., 2019). Heterogeneity was assessed using the I2 statistic (Higgins et al., 2019). Publication bias was assessed by funnel plot asymmetry, tested with an adapted Egger’s test (Pustejovsky and Rodgers, 2019). Sensitivity analysis was performed by removing statistical outliers and by removing studies with p-value-based imputed SMDs. The alpha level for statistical significance for all analyses was set to *p* < 0.05. The results of each meta-analysis were visualised in a forest plot and an overview plot containing the pooled SMD and PI of each meta-analysis.

### 2.7 Deviations from the study protocol

We intended to perform multilevel random-effects meta-analyses with neuroimaging information and connectivity matrix information included as covariates. Due to substantial missing data in study characteristics, we opted to forgo the multilevel meta-regression approach in favour of random-effect meta-analyses. Additionally, we intended to conduct subgroup analyses based on pain duration and psychological comorbidities, but this was not feasible due to the same reason.

## 3 Results

### 3.1 Study selection and characteristics

**Figure 1:**
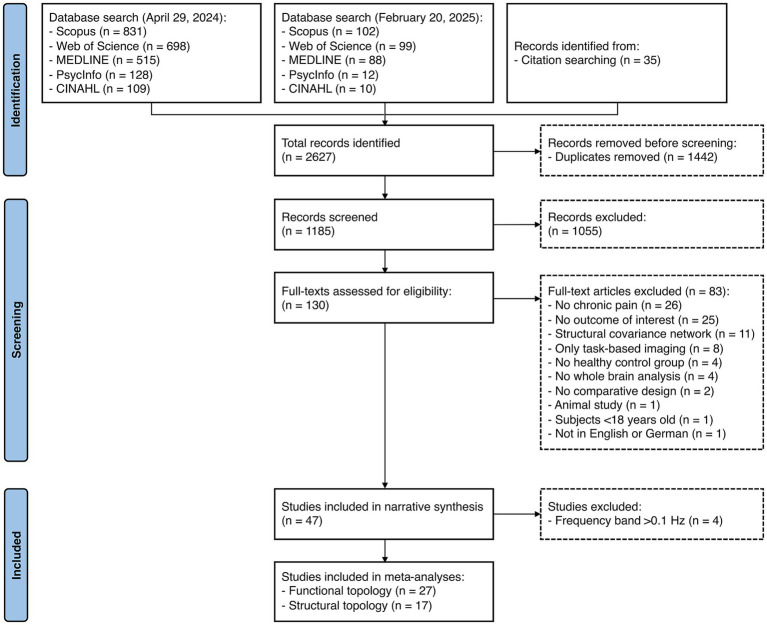
PRISMA-flowchart of study selection.

Overall, 1,185 articles were screened at the title/abstract phase, and 130 full texts were assessed (Figure 1). Finally, 47 studies were included in qualitative synthesis (Balenzuela et al., 2010; Liu et al., 2011, 2012, 2013, 2017, 2018; Zhang et al., 2014, 2017; Zhang et al., 2022b; Zhang et al., 2022a; Mansour et al., 2016; Pijnenburg et al., 2016; Wu et al., 2016, 2020; Li et al., 2017; Wada et al., 2017; Huang et al., 2019, 2021; Kaplan et al., 2019; Shi et al., 2019, 2020; Ta Dinh et al., 2019; Tu et al., 2019, 2023; De Pauw et al., 2020; Nieboer et al., 2020; Barroso et al., 2021; Dai et al., 2021; Duan et al., 2021; Fauchon et al., 2021; Kurokawa et al., 2021; Larkin et al., 2021; Silvestro et al., 2021; Chao et al., 2022; Lin et al., 2022; Wang et al., 2022, 2024; Lee et al., 2023; Qiu et al., 2023; Yang et al., 2023, 2024, 2025; Kang et al., 2024; Mao et al., 2024; Matoso et al., 2024; Mei et al., 2024; Zhou et al., 2024). Excluded studies with the reason for exclusion are provided in Appendix 3. Two studies examined both functional and structural topology (Zhang et al., 2022a; Yang et al., 2024).

#### 3.1.1 Functional topology studies

Overall, 32 studies examined functional brain topology. A qualitative summary of the included functional topology studies is provided in Table 2. Notably, a considerable number of studies did not report basic population characteristics such as sex, exact pain duration or pain intensity. Twenty-eight studies used rs-fMRI, three used rs-MEG, and one used rs-EEG. Of the 27 studies using an atlas-based parcellation approach, 15 (56%) chose a version of the AAL atlas. The existence of psychological comorbidities was an exclusion criterion in 20 (63%) studies, 15 (47%) studies assessed psychological comorbidities using patient-reported outcome measures (eight [25%] studies only in the chronic pain group), and eight (25%) studies did not report them at all. A detailed overview of all functional topology studies is presented in Appendix 4.

**Table 2:** Summary of study characteristics.

	Functional topology studies (k = 32)	Structural topology studies (k = 17)
Population characteristics		
Study size (n)	24–185	25–185
Pain type (k)	CPP (k = 17) CSP (k = 15) Mixed (k = 1)	CPP (k = 13) CSP (k = 4)
Sex (% of ♂)	0–100% *Missing (k = 4)*	0–100% *Missing (k = 2)*
Mean age (years)	22–73 *Missing (k = 1)*	22–60
Pain duration (months)	5–228 Missing (k = 10)*	35–241 Missing (k = 3)*
Pain intensity (NRS)	3.7–8.2 *Missing (k = 7)*	4.1–8.1 *Missing (k = 2)*
Imaging characteristics		
Imaging modality	rs-fMRI (k = 28) rs-MEG (k = 3) rs-EEG (k = 1)	dMRI (k = 17)
MRI-specific characteristics		
MRI field strength	3 T (k = 26) 1.5 T (k = 1)	3 T (k = 16) 1.5 T (k = 1)
Voxel dimensions	Isotropic (k = 9) Anisotropic (k = 18) 2–8 mm	Isotropic (k = 9) Anisotropic (k = 8) 1.1–3.3 mm
Modality-specific	Resting-state frequency: 0.008–0.1 Hz (*one exception with low-passfilter of 0.15 Hz*)	Unique diffusion gradient directions: 30–124 b-value: 1,000–3,000 Tractography: Deterministic (k = 7) Probabilistic (k = 7) *Missing (k = 3)*
Network characteristics		
Parcellation approach	Atlas-based (k = 27) ICA-based (k = 2) Voxel-based (k = 2) Channel-based (k = 1)	Atlas-based (k = 17)
Node size (n)	8–400 (*Two exceptions with* 2020 and 5,828)	15–300
Network thresholding	Density (k = 27) Absolute (k = 1) MST (k = 1) None (k = 1) *Missing (k = 2)*	Density (k = 5) Absolute (k = 1) Statistical sig. (k = 1) None (k = 5) *Missing (k = 5)*
Edge type	Binary (k = 20) Weighted (k = 5) *Missing (k = 7)*	Binary (k = 7) Weighted (k = 8) *Missing (k = 3)*
Edge metric	Full correlation (k = 25) Partial correlation (k = 2) Other (k = 5)	SLC (k = 11) FA (k = 6)
Network properties (n)	21	14

*No exact pain duration was reported but inclusion criteria specified pain duration of >3 months. CPP, Chronic primary pain. CSP, Chronic secondary pain. dMRI, Diffusion-weighted magnetic resonance imaging. FA, Fractional anisotropy. ICA, Independent component analysis. MST, Minimum spanning tree. NRS, Numeric rating scale. rs-EEG, Resting-state electroencephalogram. rs-fMRI, Resting-state functional magnetic resonance imaging. rs-MEG, Resting-state magnetoencephalography. SLC, Streamline count.

#### 3.1.2 Structural topology studies

Structural brain topology was investigated by 17 studies. A qualitative summary of the included structural topology studies is provided in Table 1. Notably, a considerable number of studies did not report basic population characteristics such as sex, exact pain duration or pain intensity. Twelve (71%) studies employed a version of the AAL atlas. A single-shell dMRI acquisition protocol was used in 15 (88%) of included studies. The existence of psychological comorbidities was an exclusion criterion in six (35%) studies, seven (41%) studies assessed psychological comorbidities using patient-reported outcome measures (five [29%] only in the chronic pain group), and seven (41%) studies did not report psychological comorbidities at all. A detailed overview of all structural topology studies is presented in Appendix 4.

#### 3.1.3 Risk of bias

**Figure 2:**
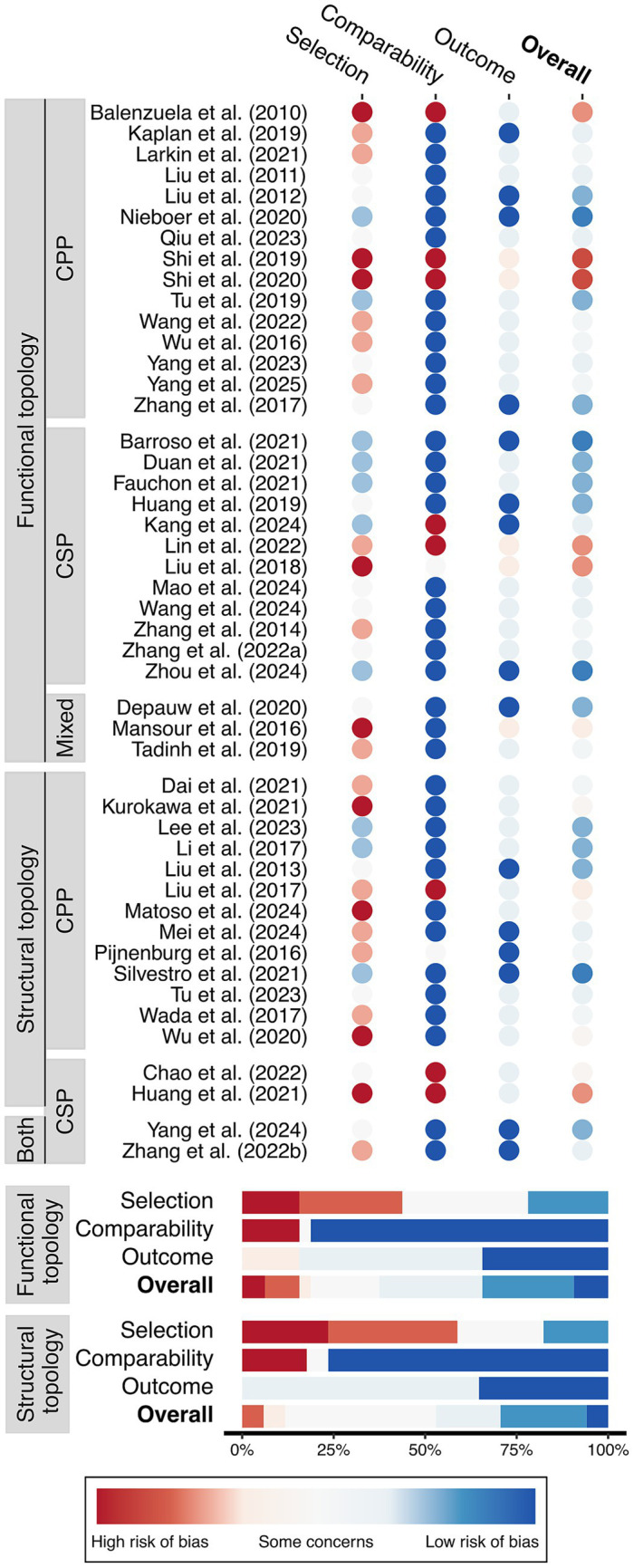
Risk of bias assessment grouped by functional and structural topology, chronic primary pain (CPP), and chronic secondary pain (CSP).

The results of the risk of bias assessment are illustrated in Figure 2. Among the 32 studies that assessed functional brain topology, 11 (34%) were deemed to have low risk of bias, 16 (50%) had some concerns, and five (16%) were classified as having a high risk of bias. Of the 17 studies that examined structural brain topology, five (29%) scored low risk of bias, 11 (65%) had some concerns, and one (6%) had high risk of bias. When examining domain-specific risk of bias, 14 (44%) and 10 (59%) of functional and structural topology studies exhibited a high risk of bias in participant selection.

### 3.2 Meta-analysis

#### 3.2.1 Data handling

For at least one outcome, the mean and standard deviation were extracted directly in 16 studies (Zhang et al., 2014; Liu et al., 2017, 2018; Kaplan et al., 2019; Wu et al., 2020; Dai et al., 2021; Fauchon et al., 2021; Huang et al., 2021; Kurokawa et al., 2021; Chao et al., 2022; Wang et al., 2022, 2024; Lee et al., 2023; Yang et al., 2023, 2024; Mao et al., 2024) WebPlotDigitizer was used in 24 studies (Balenzuela et al., 2010; Liu et al., 2011, 2012, 2013; Mansour et al., 2016; Pijnenburg et al., 2016; Li et al., 2017; Zhang et al., 2017, 2022a,b; Huang et al., 2019; Shi et al., 2019; De Pauw et al., 2020; Barroso et al., 2021; Duan et al., 2021; Fauchon et al., 2021; Larkin et al., 2021; Lin et al., 2022; Tu et al., 2023; Kang et al., 2024; Matoso et al., 2024; Mei et al., 2024; Zhou et al., 2024; Yang et al., 2025), and the standard deviation was imputed in five studies (Balenzuela et al., 2010; Shi et al., 2019; Silvestro et al., 2021; Zhang et al., 2022b; Kang et al., 2024). Pooling of multiple groups was performed in 10 studies (Liu et al., 2011; Mansour et al., 2016; De Pauw et al., 2020; Wu et al., 2020; Dai et al., 2021; Duan et al., 2021; Fauchon et al., 2021; Mei et al., 2024; Yang et al., 2024; Zhou et al., 2024). Two of these studies included both a chronic primary and secondary pain group, which were handled separately for the subgroup analysis (De Pauw et al., 2020; Mansour et al., 2016). In 40 studies, the SMD was computed based on the mean and standard deviation for at least one outcome (Balenzuela et al., 2010; Liu et al., 2011, 2012, 2013, 2017, 2018; Zhang et al., 2014, 2017, 2022a,b;Mansour et al., 2016; Pijnenburg et al., 2016; Li et al., 2017; Huang et al., 2019, 2021; Kaplan et al., 2019; Shi et al., 2019; De Pauw et al., 2020; Wu et al., 2020; Barroso et al., 2021; Dai et al., 2021; Duan et al., 2021; Fauchon et al., 2021; Kurokawa et al., 2021; Larkin et al., 2021; Silvestro et al., 2021; Chao et al., 2022; Lin et al., 2022; Wang et al., 2022, 2024; Lee et al., 2023; Tu et al., 2023; Yang et al., 2023, 2024, 2025; Kang et al., 2024; Mao et al., 2024; Matoso et al., 2024; Mei et al., 2024; Zhou et al., 2024). In four studies, the SMD was estimated based on the *p*-value (Wada et al., 2017; Shi et al., 2019; Silvestro et al., 2021; Tu et al., 2023), and in one study, the *F*-value was used (Tu et al., 2019). The SMD could not be estimated for at least one outcome in four studies due to insufficient reporting (Balenzuela et al., 2010; Shi et al., 2020; Barroso et al., 2021; Lin et al., 2022). The authors were contacted in 12 cases, with a response rate of 8% (*n* = 1).

#### 3.2.2 Functional topology

**Figure 3:**
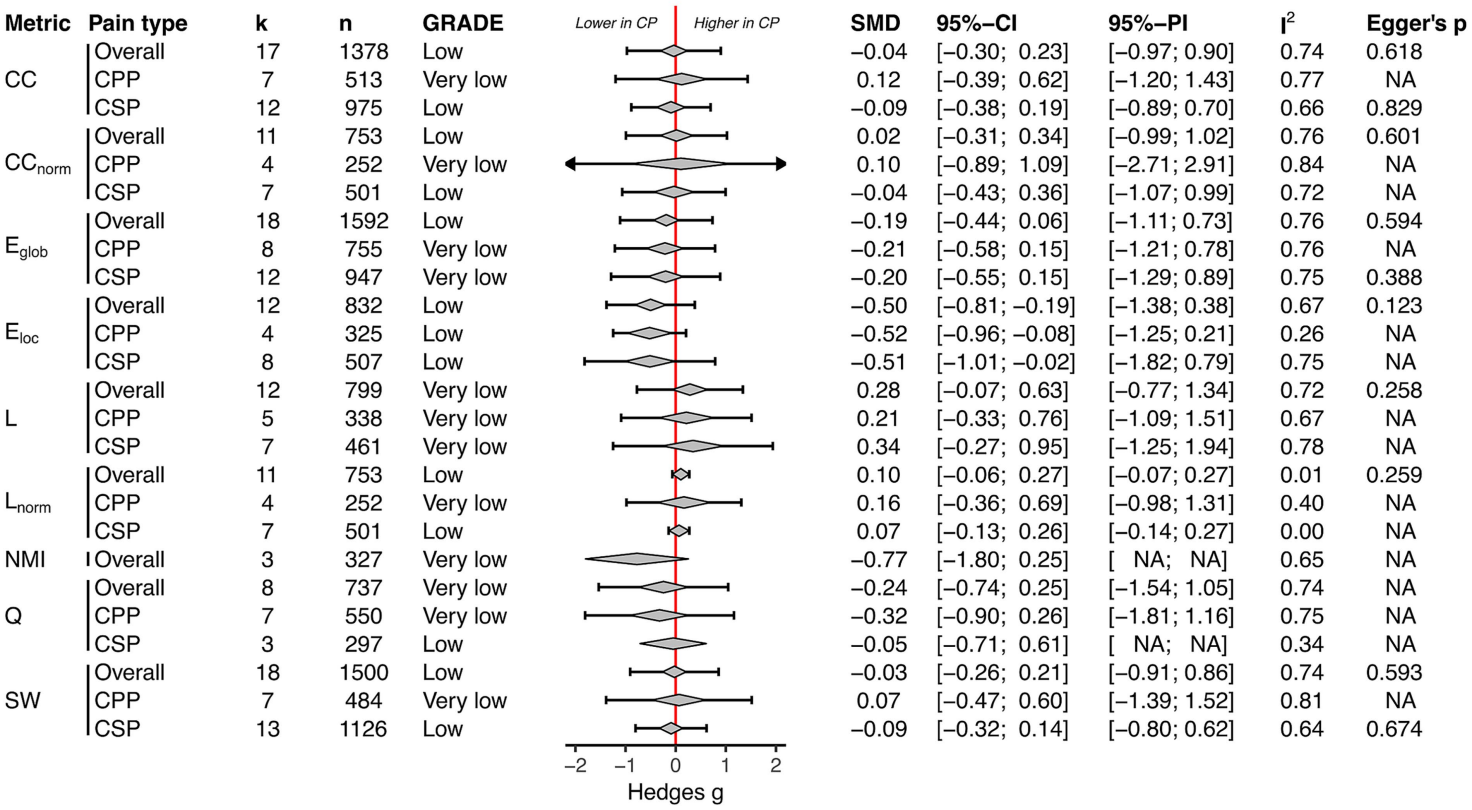
Functional topology: Summary of all meta-analyses ordered by global network property (clustering coefficient [CC]; normalised clustering coefficient [CC_norm_]; global efficiency [E_glob_]; local efficiency [E_loc_]; characteristic path length [L]; normalised characteristic path length [Lnorm]; normalised mutual information [NMI]; modularity [Q]; small-worldness [SW]) and pain type (overall; chronic primary pain [CPP]; chronic secondary pain [CSP]). The diamond depicts the pooled SMD and the error bar its 95% prediction interval; CP, Chronic pain.

Meta-analysis was eligible for nine unique global network properties of functional topology (across 27 rs-fMRI studies). A qualitative synthesis of studies excluded from the meta-analysis is presented in Appendix 5. There was low-certainty evidence that chronic pain patients showed lower local efficiency in functional whole-brain topology (Figure 3). Additionally, there was low to very low-certainty evidence that chronic pain patients exhibited no difference in functional whole-brain topology assessed by clustering coefficient, normalised clustering coefficient, global efficiency, characteristic path length, normalised characteristic path length, normalised mutual information, modularity and small-worldness (Figure 3). The heterogeneity in overall chronic pain meta-analysis was high, ranging from 67 to 76%, except for the normalised characteristic path length, with 1%. Subgroup analysis showed similar results, with low-certainty evidence of lower local efficiency in chronic primary pain and chronic secondary pain compared to healthy controls (Figure 3). All accompanying forest plots are presented in Appendix 6. The results were robust to outlier removal sensitivity analysis (Appendix 6). When removing studies with p-value-based imputed SMDs, the significant difference in local efficiency between chronic primary pain and healthy controls disappeared (SMD, −0.51; 95%-CI: −1.47, 0.45; Appendix 6). All other results were robust to this sensitivity analysis (Appendix 6).

#### 3.2.3 Structural topology

**Figure 4:**
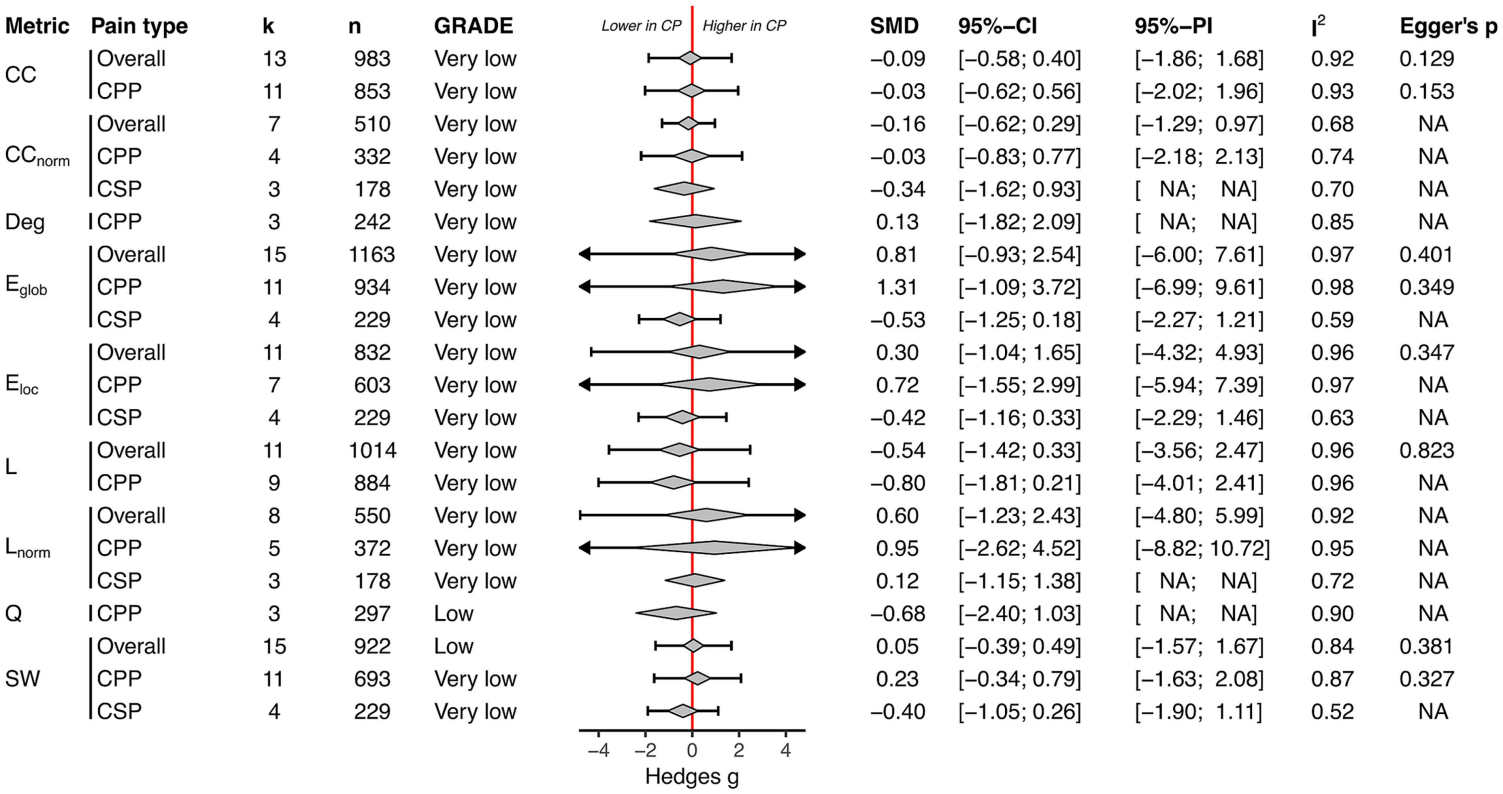
Structural topology: Summary of all meta-analyses ordered by global network property (clustering coefficient [CC]; normalised clustering coefficient [CC_norm_]; degree [deg]; global efficiency [E_glob_]; local efficiency [E_loc_]; characteristic path length [L]; normalised characteristic path length [Lnorm]; modularity [Q]; small-worldness [SW]) and pain type (Overall, chronic primary pain [CPP], chronic secondary pain [CSP]). The diamond depicts the pooled SMD and the error bar its 95% prediction interval; CP, Chronic pain.

Meta-analysis was eligible for nine unique global network properties of structural topology (across 17 dMRI studies). There was low to very low-certainty evidence that chronic pain patients showed no difference in structural whole-brain topology assessed by average degree, clustering coefficient, normalised clustering coefficient, global efficiency, local efficiency, characteristic path length, normalised characteristic path length, modularity, and small-worldness (Figure 4). The heterogeneity in chronic pain overall meta-analyses was substantial, ranging from 68 to 97%. Subgroup analysis also revealed no alterations in structural brain topology in chronic primary and secondary pain (Figure 4). In outlier-removal-sensitivity analysis, the results remained non-significant (Appendix 7). The results were robust when removing studies with p-value-based imputed SMDs (Appendix 7).

## 4 Discussion

The primary aim of this study was to quantitatively synthesise alterations in functional and structural connectivity-based global network properties of whole-brain topology in chronic pain. There was low-certainty evidence that chronic pain patients showed impairments in local efficiency of resting-state functional whole-brain topology, and low to very low-certainty evidence that structural whole-brain topology was not altered in chronic pain.

### 4.1 Lower local efficiency in resting-state functional network in chronic pain

**Figure 5:**
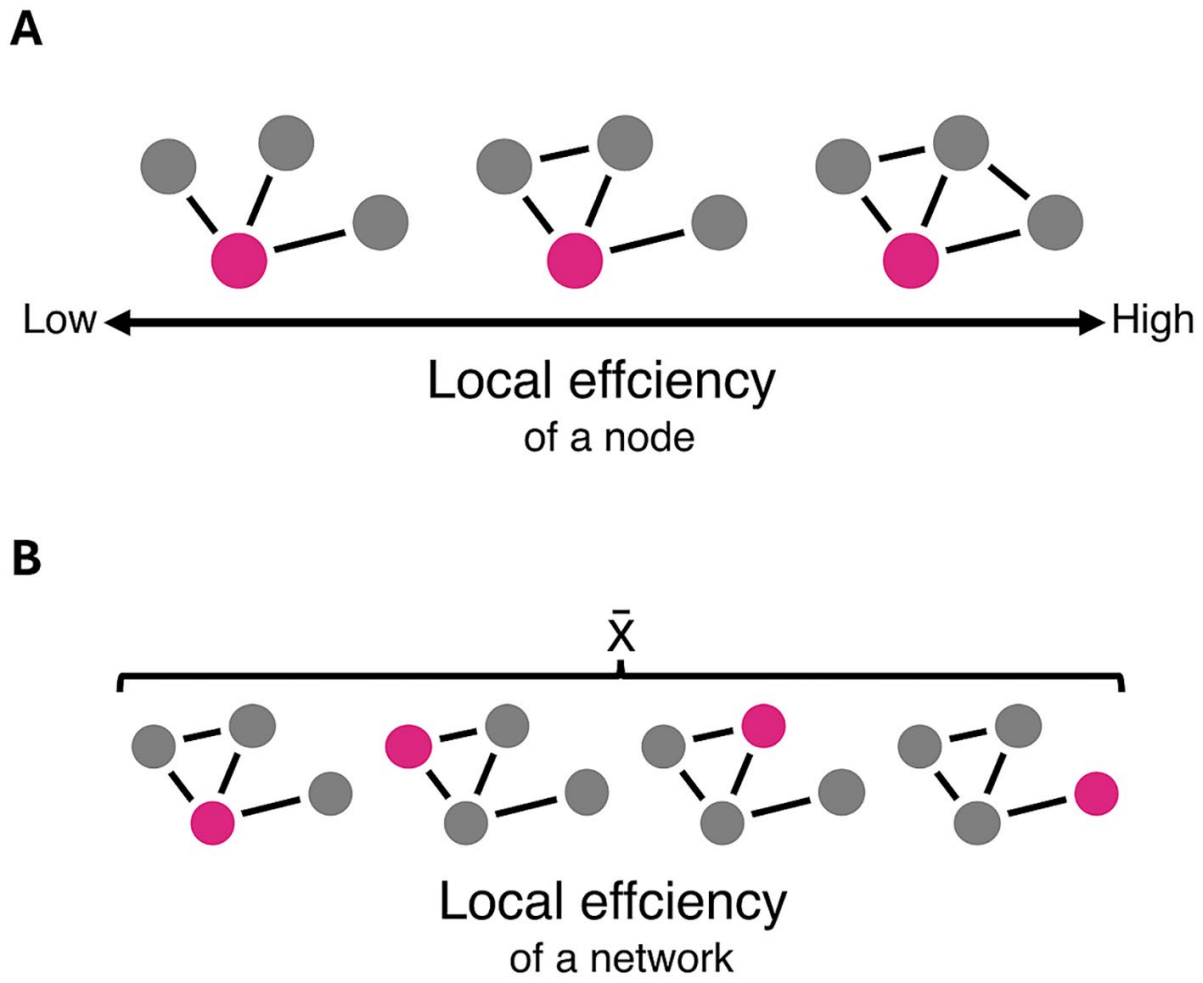
Schematic representation of local efficiency. **(A)** Local efficiency of a node is increased as the number of connections between its neighbouring nodes increases. **(B)** Local efficiency as a global network property is determined by the average local efficiency of all nodes within a given network.

Local efficiency quantifies how effectively information is transferred within local neighbourhoods of a network (Figure 5). It is therefore a measure of segregation, reflecting the brain’s capacity for specialised processing within densely interconnected groups of brain regions (Rubinov and Sporns, 2010). Patients with chronic pain showed lower local efficiency in their resting-state functional network, indicating reduced local specialisation and suggesting a shift towards greater global integration. Both chronic primary pain and chronic secondary pain exhibited impairments in local efficiency, suggesting that chronic pain, regardless of its origin, is associated with similar reorganisation of functional whole-brain topology.

In healthy individuals, the resting state is dominated by activity in the default mode network (DMN), associated with self-referential thought. A recent meta-analysis of resting-state fMRI studies concluded that patients with chronic pain exhibit altered intra-DMN connectivity and increased connectivity between DMN and somatosensory regions (Fiúza-Fernandes et al., 2025). This pattern further supports a shift towards global integration through stronger interconnectedness of self-referential processes and pain processing.

Impairments in local efficiency of the functional whole-brain network have also been reported in a meta-analysis of patients with depression (Xu et al., 2021), a common comorbidity in chronic pain (De La Rosa et al., 2024). Due to inconsistent reporting of psychological comorbidities in the included studies, we were unable to determine their influence on functional topology alterations in chronic pain. Therefore, it is unclear whether local efficiency impairments are characteristic of chronic pain, are conditional on the presence of psychological comorbidities such as depression, or are a shared neural principle across conditions.

### 4.2 No structural whole-brain topology alterations in chronic pain

Our meta-analyses concluded that the structural whole-brain topology remains unaltered in chronic pain. This contrasts with neurological diseases with substantial anatomical changes, such as epilepsy, Parkinson’s disease, or traumatic brain injury, which have demonstrated impairments in structural whole-brain topology (Imms et al., 2019; Slinger et al., 2022; Zuo et al., 2023). Interestingly, dMRI-based alterations of various white matter bundles have been documented in chronic pain, including the corpus callosum, thalamic radiations, internal capsule and corona radiata (Bautin et al., 2025). However, these microstructural changes do not necessarily affect the global organisation of the structural connectome, suggesting preserved large-scale brain architecture despite local white matter disruptions.

### 4.3 Heterogeneity and risk of bias

The heterogeneity was with I^2^ values of more than 50% in most meta-analyses, and wide prediction intervals, considered to be high. Although no meta-regression was performed to evaluate heterogeneity, the qualitative synthesis showed a wide range in population and methodological characteristics of the included studies. For example, included studies investigated different chronic pain syndromes, including headache, orofacial pain, visceral pain, musculoskeletal pain, widespread pain, complex regional pain syndromes and neuropathic pain. Notably, half of the structural topology studies and about one-third of functional topology studies examined migraine, indicating a bias in the meta-analyses.

The methodological characteristics of the included studies reflect the current lack of a gold standard in graph-theoretical brain network analysis (Fornito et al., 2013; Bahrami et al., 2023). Differences in parcellation scheme, network thresholding, and edge definition may have influenced the results of our meta-analysis. While several global network properties remain robust across different parcellation schemes at a consistent resolution (number of nodes), altering the parcellation resolution significantly impacts these global metrics (Zalesky et al., 2010). Since the number of nodes (84–264) of most studies was on a similar scale, parcellation was less likely to influence the results. However, it must be noted that in functional networks, larger parcellation units yield smoother, less specific time courses, which in turn reduce the specificity of the resulting network topology (De Reus and Van Den Heuvel, 2013). Different thresholding methods are applied to networks to account for spurious connections, but they introduce specific limitations (van Wijk et al., 2010). Although most included studies employed density-based thresholding, which retains a specific percentage of the strongest connections, the range of the used thresholds varied widely, adding heterogeneity to the meta-analyses as the choice of a density threshold influences the graph measures (van Wijk et al., 2010). Additionally, structural topology studies showed higher heterogeneity in network binarisation and edge metric, adding further variability, while functional topology studies showed higher concordance in these characteristics. Many rs-fMRI and some dMRI studies acquired anisotropic voxels, potentially introducing anatomical inaccuracies, partial volume effects, and other directional biases (Mulder et al., 2019). Most structural topology studies used the simple DTI model with a one-shell diffusion acquisition scheme with 30 to 64 directions, yielding high residuals in the tensor model. Future studies should embrace the advancements made in dMRI acquisition and processing over the last 15 years by utilising multi-shell acquisitions, which enable state-of-the-art dMRI analysis (Bautin et al., 2025).

The risk of bias assessment of this review indicates a considerable risk in the selection of study participants, a common pitfall of cross-sectional studies. Future studies should improve this by clearly defining their control group and by investigating more representative study samples. Strikingly, many studies showed poor reporting, missing basic population and methodological information such as sex, exact pain duration, pain intensity, edge type, network thresholding or tractography algorithm. Additionally, some studies lacked sufficient outcome data for cross-study pooling. Overall, the methodological heterogeneity and concerning reporting quality complicate the interpretation of cross-study comparisons of global network properties and call for standardisation in graph theoretical analysis of brain networks.

### 4.4 Limitations

The findings of this review should be considered with several methodological limitations in mind. First, as the meta-analyses only included functional topology studies investigating low-frequency fluctuations (≤ 0.1 Hz), no quantitative statement about other frequency bands (investigated in four studies) can be made. Second, four studies were excluded from the meta-analyses because their results could not be extracted due to poor reporting. As their results showed no alterations in brain topology, they would likely not alter the overall results of this review. Third, not all reported global network properties could be pooled due to limitations in the number of studies investigating them, as not all studies reported all metrics. Fourth, the review excluded studies investigating structural covariance networks as they do not analyse white matter topology but analyse structural topology based on the correlation of morphology parameters of grey matter regions. It remains unclear whether this approach can identify structural whole-brain topology alterations in chronic pain. Additionally, systematic differences in brain topology alteration may exist among different pain diagnoses. Pooling data across all diagnoses may have introduced sufficient variance to obscure any difference between healthy controls and chronic pain patients.

### 4.5 Implications for clinic and research

Our meta-analysis showed that impairments in local efficiency within the resting-state whole-brain network are associated with both chronic primary and secondary pain diagnoses. However, it remains unclear whether the functional topology reorganisation is a consequence of chronic pain or if it contributes to its development. Future studies may focus on determining the time course of functional topology reorganisation, its modulation by therapeutic interventions and consider psychological comorbidities as a possible influencing factor.

While the whole-brain approach captures essential characteristics of the entire macroscale brain organisation, it may lack sensitivity to detect subtle disease-related changes in network topology. Investigations of subnetworks such as large-scale brain networks (Uddin et al., 2019), or the ‘pain matrix’ (Legrain et al., 2011) could reveal structural and functional topology alterations confined to specific subnetworks previously masked by the whole-brain approach. Additionally, future studies could use further global network properties such as the hub disruption index, which captures changes at the nodal level, shows high reliability and sensitivity (Termenon et al., 2016), and has already been used in some pain studies (Huang et al., 2019; De Pauw et al., 2020; Barroso et al., 2021).

## 5 Conclusion

This is the first study to quantitatively synthesise connectivity-based global network properties of functional and structural brain topology in chronic pain. There was low-certainty evidence that chronic pain patients showed impairments in local efficiency of resting-state functional whole-brain topology, and low to very low-certainty evidence that structural whole-brain topology was not altered in chronic pain. This indicates that functional but not anatomical whole-brain topological reorganisation is involved in the pathophysiology of chronic pain. This review highlights the need for standardisation in graph-theoretical brain network analyses and reporting of methods and results. Additionally, future studies should consider psychological comorbidities and expand the analyses to specific subnetworks when investigating brain topology in chronic pain.

## Author’s note

This work is part of the PhD project of L. Butry.

## Data Availability

Publicly available datasets were analysed in this study. This data can be found at: https://osf.io/y27qc/.
