# Supplementary material for: Brain network properties in chronic pain—a systematic review and meta-analysis of graph-based connectivity metrics
Source: Front Neurosci. 2025 Nov 18;19:1672542. doi: 10.3389/fnins.2025.1672542 (PMC12669219; doi:10.3389/fnins.2025.1672542)
Supplement: Supplementary file 1 [file Data_Sheet_1.pdf]

Supplementary Material

Table of contents

1 Appendix 1 – Search strategy 2

2 Appendix 2 – Additional methods 7

2.1 Formulas used in data extraction and GRADE criteria 7

2.2 Data extraction with 'WebPlotDigitizer' 8

2.3 Newcastle-Ottawa Scale – Coding manual adapted for the 'NET-PAIN' review 8

3 Appendix 3 – Studies excluded at the full-text phase with reasons 10

4 Appendix 4 – Study characteristics of included studies 13

5 Appendix 5: Results not included in meta-analyses 18

6 Appendix 6 – Results of functional brain topology (FT) meta-analysis 18

6.1 Forest plots of FT – Overall 18

6.2 Forest plots of FT subgroup analysis: Chronic primary pain (CPP) 21

6.3 Forest plots of FT subgroup analysis: Chronic secondary pain (CSP) 24

6.4 Sensitivity analysis of functional topology meta-analysis 26

7 Appendix 7 – Results of structural brain topology (ST) meta-analysis 28

7.1 Forest plots of ST – Overall 28

7.2 Forest plots of ST subgroup analysis: Chronic primary pain (CPP) 30

7.3 Forest plots of ST subgroup analysis: Chronic secondary pain (CSP) 32

7.4 Sensitivity analysis of structural topology meta-analysis 34

8 PRISMA Checklist 36

1 Appendix 1 – Search strategy

Table 1.1 MEDLINE via PubMed

Search	Query
**#1**	(pain[Mesh] OR pain*[tiab] OR "chronic pain"[tiab] OR ache[tiab] OR headache[tiab] OR migraine[tiab] OR polyneuropathy[tiab] OR myalgia[tiab] OR fibromyalgia [tiab] OR WAD[tiab] OR "whiplash associated disorder"[tiab] OR "temporomandibular disorder"[tiab] OR osteoarthritis[tiab] OR arthritis[tiab] OR arthralgia[tiab] OR neuralgia[tiab] OR neuropathy[tiab] OR radiculopathy[tiab] OR stenosis[tiab] OR spondylolisthesis[tiab] OR spondylosis[tiab] OR "disc hernia"[tiab] OR "disc prolapse"[tiab] OR sciatica[tiab] OR lumbago[tiab] OR scoliosis[tiab] OR "sulcus ulnaris syndrome"[tiab] OR "tunnel syndrome"[tiab] OR "frozen shoulder"[tiab] OR "adhesive capsulitis"[tiab] OR "temporomandibular joint disorder"[tiab] OR "burning mouth syndrome"[tiab] OR "spinal cord injur*"[tiab] OR CRPS[tiab] OR "irritable bowel syndrome"[tiab] OR IBS[tiab] OR "inflammatory bowel disease"[tiab] OR IBD[tiab] OR crohn[tiab] OR colitis[tiab] OR cystitis[tiab] OR endometriosis[tiab] OR prostatitis[tiab] OR pancreatitis[tiab] OR angina[tiab] OR "herpes zoster"[tiab] OR shingles[tiab] OR dyspepsia[tiab] OR adenomyosis[tiab] OR "pelvic congestion syndrome"[tiab] OR vulvodynia[tiab] OR "gastroesophageal reflux disease"[tiab] OR GERD[tiab] OR "peripheral artery diseas*"[tiab] OR PAD[tiab] OR burn*[tiab])
**#2**	(connectome[Mesh] OR connectome[tiab] OR connectivity[tiab] OR brain[tiab] OR topolog*[tiab] OR "functional network*"[tiab] OR "functional architect*"[tiab] OR "functional organi*"[tiab] OR "structural network*"[tiab] OR "structural architect*"[tiab] OR "structural organi*"[tiab] OR "network architect*"[tiab] OR "network organi*"[tiab] OR "magnetic resonance imaging"[Mesh] OR MRI[tiab] OR fMRI[tiab] OR "functional MRI"[tiab] OR rs-fMRI[tiab] OR rsfmri[tiab] OR DTI[tiab] OR DKI[tiab] OR "diffusion tensor imaging"[tiab] OR "diffusion kurtosis imaging"[tiab] OR tractography[tiab] OR EEG[tiab] OR electroencephalography[tiab] OR electroencephalography[Mesh] OR MEG[tiab] OR magnetoencephalogram[tiab] OR "brain imaging"[tiab])
**#3**	(graph[tiab] OR "network propert*"[tiab] OR "network characteristic*"[tiab] OR "network analys*"[tiab] OR "network theoretical analysis"[tiab] OR "global network"[tiab] OR "characteristic path length"[tiab] OR "global efficiency"[tiab] OR "clustering coefficient"[tiab] OR transitivity[tiab] OR "local efficiency"[tiab] OR modularity[tiab] OR "average degree"[tiab] OR "average strength"[tiab] OR "average eccentricity"[tiab] OR "small world*"[tiab] OR "rich club*"[tiab])
**#4**	#1 AND #2 AND #3
**Initial search (Database start – 2024-04-29):**<br>**Second search (2024-04-29 – 2025-02-20):**<br>**Overall:**	515 Hits<br>88 Hits<br>**603 Hits**

Table 1.2 SCOPUS (Limted to articles)

Search	Query
**#1**	(INDEXTERMS(pain) OR TITLE-ABS(pain*) OR TITLE-ABS("chronic pain") OR TITLE-ABS(ache) OR TITLE-ABS(headache) OR TITLE-ABS(migraine) OR TITLE-ABS(polyneuropathy) OR TITLE-ABS(myalgia) OR TITLE-ABS(fibromyalgia) OR TITLE-ABS(WAD) OR TITLE-ABS("whiplash associated disorder") OR TITLE-ABS("temporomandibular disorder") OR TITLE-ABS(osteoarthritis) OR TITLE-ABS(arthritis) OR TITLE-ABS(arthralgia) OR TITLE-ABS(neuralgia) OR TITLE-ABS(neuropathy) OR TITLE-ABS(radiculopathy) OR TITLE-ABS(stenosis) OR TITLE-ABS(spondylolisthesis) OR TITLE-ABS(spondylosis) OR TITLE-ABS("disc hernia") OR TITLE-ABS("disc prolapse") OR TITLE-ABS(sciatica) OR TITLE-ABS(lumbago) OR TITLE-ABS(scoliosis) OR TITLE-ABS("sulcus ulnaris syndrome") OR TITLE-ABS("tunnel syndrome") OR TITLE-ABS("frozen shoulder") OR TITLE-ABS("adhesive capsulitis") OR TITLE-ABS("temporomandibular joint disorder") OR TITLE-ABS("burning mouth syndrome") OR TITLE-ABS("spinal cord injur*") OR TITLE-ABS(CRPS) OR TITLE-ABS("irritable bowel syndrome") OR TITLE-ABS(IBS) OR TITLE-ABS("inflammatory bowel disease") OR TITLE-ABS(IBD) OR TITLE-ABS(crohn) OR TITLE-ABS(colitis) OR TITLE-ABS(cystitis) OR TITLE-ABS(endometriosis) OR TITLE-ABS(prostatitis) OR TITLE-ABS(pancreatitis) OR TITLE-ABS(angina) OR TITLE-ABS("herpes zoster") OR TITLE-ABS(shingles) OR TITLE-ABS(dyspepsia) OR TITLE-ABS(adenomyosis) OR TITLE-ABS("pelvic congestion syndrome") OR TITLE-

	ABS(vulvodynia) OR TITLE-ABS("gastroesophageal reflux disease") OR TITLE-ABS(GERD) OR TITLE-ABS("peripheral artery diseas*") OR TITLE-ABS(PAD) OR TITLE-ABS(burn*))
**#2**	(INDEXTERMS(connectome) OR TITLE-ABS(connectome) OR TITLE-ABS(connectivity) OR TITLE-ABS(brain) OR TITLE-ABS(topolog*) OR TITLE-ABS("functional network*") OR TITLE-ABS("functional architect*") OR TITLE-ABS("functional organi*") OR TITLE-ABS("structural network*") OR TITLE-ABS("structural architect*") OR TITLE-ABS("structural organi*") OR TITLE-ABS("network architect*") OR TITLE-ABS("network organi*") OR INDEXTERMS("magnetic resonance imaging") OR TITLE-ABS(MRI) OR TITLE-ABS(fMRI) OR TITLE-ABS("functional MRI") OR TITLE-ABS(rs-fMRI) OR TITLE-ABS(rsfmri) OR TITLE-ABS(DTI) OR TITLE-ABS(DKI) OR TITLE-ABS("diffusion tensor imaging") OR TITLE-ABS("diffusion kurtosis imaging") OR TITLE-ABS(tractography) OR TITLE-ABS(EEG) OR TITLE-ABS(electroencephalography) OR INDEXTERMS(electroencephalography) OR TITLE-ABS(MEG) OR TITLE-ABS(magnetoencephalogram) OR TITLE-ABS("brain imaging"))
**#3**	(TITLE-ABS(graph) OR TITLE-ABS("network propert*") OR TITLE-ABS("network characteristic*") OR TITLE-ABS("network analys*") OR TITLE-ABS("network theoretical analysis") OR TITLE-ABS("global network") OR TITLE-ABS("characteristic path length") OR TITLE-ABS("global efficiency") OR TITLE-ABS("clustering coefficient") OR TITLE-ABS(transitivity) OR TITLE-ABS("local efficiency") OR TITLE-ABS(modularity) OR TITLE-ABS("average degree") OR TITLE-ABS("average strength") OR TITLE-ABS("average eccentricity") OR TITLE-ABS("small world*") OR TITLE-ABS("rich club*"))
**#4**	#1 AND #2 AND #3
**Initial search (Database start – 2024-04-29):**<br>**Second search (2024-04-29 – 2025-02-20):**<br>**Overall:**	831 Hits<br>102 Hits<br>**933 Hits**

Table 1.3 Web of Science

Search	Query
**#1**	(ALL=pain OR (TI=pain* OR AB=pain*) OR (TI="chronic pain" OR AB="chronic pain") OR (TI=ache OR AB=ache) OR (TI=headache OR AB=headache) OR (TI=migraine OR AB=migraine) OR (TI=polyneuropathy OR AB=polyneuropathy) OR (TI=myalgia OR AB=myalgia) OR (TI=fibromyalgia OR AB=fibromyalgia) OR (TI=WAD OR AB=WAD) OR (TI="whiplash associated disorder" OR AB="whiplash associated disorder") OR (TI="temporomandibular disorder" OR AB="temporomandibular disorder") OR (TI=osteoarthritis OR AB=osteoarthritis) OR (TI=arthritis OR AB=arthritis) OR (TI=arthralgia OR AB=arthralgia) OR (TI=neuralgia OR AB=neuralgia) OR (TI=neuropathy OR AB=neuropathy) OR (TI=radiculopathy OR AB=radiculopathy) OR (TI=stenosis OR AB=stenosis) OR (TI=spondylolisthesis OR AB=spondylolisthesis) OR (TI=spondylosis OR AB=spondylosis) OR (TI="disc hernia" OR AB="disc hernia") OR (TI="disc prolapse" OR AB="disc prolapse") OR (TI=sciatica OR AB=sciatica) OR (TI=lumbago OR AB=lumbago) OR (TI=scoliosis OR AB=scoliosis) OR (TI="sulcus ulnaris syndrome" OR AB="sulcus ulnaris syndrome") OR (TI="tunnel syndrome" OR AB="tunnel syndrome") OR (TI="frozen shoulder" OR AB="frozen shoulder") OR (TI="adhesive capsulitis" OR AB="adhesive capsulitis") OR (TI="temporomandibular joint disorder" OR AB="temporomandibular joint disorder") OR (TI="burning mouth syndrome" OR AB="burning mouth syndrome") OR (TI="spinal cord injur*" OR AB="spinal cord injur*") OR (TI=CRPS OR AB=CRPS) OR (TI="irritable bowel syndrome" OR AB="irritable bowel syndrome") OR (TI=IBS OR AB=IBS) OR (TI="inflammatory bowel disease" OR AB="inflammatory bowel disease") OR (TI=IBD OR AB=IBD) OR (TI=crohn OR AB=crohn) OR (TI=colitis OR AB=colitis) OR (TI=cystitis OR AB=cystitis) OR (TI=endometriosis OR AB=endometriosis) OR (TI=prostatitis OR AB=prostatitis) OR (TI=pancreatitis OR AB=pancreatitis) OR (TI=angina OR AB=angina) OR (TI="herpes zoster" OR AB="herpes zoster") OR (TI=shingles OR AB=shingles) OR (TI=dyspepsia OR AB=dyspepsia) OR (TI=adenomyosis OR AB=adenomyosis) OR (TI="pelvic congestion syndrome" OR AB="pelvic congestion syndrome") OR (TI=vulvodynia OR AB=vulvodynia) OR (TI="gastroesophageal reflux disease" OR AB="gastroesophageal reflux disease") OR (TI=GERD OR AB=GERD) OR (TI="peripheral artery diseas*" OR AB="peripheral artery diseas*") OR (TI=PAD OR AB=PAD) OR (TI=burn* OR AB=burn*))
**#2**	(ALL=connectome OR (TI=connectome OR AB=connectome) OR (TI=connectivity OR AB=connectivity) OR (TI=brain OR AB=brain) OR (TI=topolog* OR AB=topolog*) OR

	(TI="functional network*" OR AB="functional network*") OR (TI="functional architect*" OR AB="functional architect*") OR (TI="functional organi*" OR AB="functional organi*") OR (TI="structural network*" OR AB="structural network*") OR (TI="structural architect*" OR AB="structural architect*") OR (TI="structural organi*" OR AB="structural organi*") OR (TI="network architect*" OR AB="network architect*") OR (TI="network organi*" OR AB="network organi*") OR ALL="magnetic resonance imaging" OR (TI=MRI OR AB=MRI) OR (TI=fMRI OR AB=fMRI) OR (TI="functional MRI" OR AB="functional MRI") OR (TI=rs-fMRI OR AB=rs-fMRI) OR (TI=rsfmri OR AB=rsfmri) OR (TI=DTI OR AB=DTI) OR (TI=DKI OR AB=DKI) OR (TI="diffusion tensor imaging" OR AB="diffusion tensor imaging") OR (TI="diffusion kurtosis imaging" OR AB="diffusion kurtosis imaging") OR (TI=tractography OR AB=tractography) OR (TI=EEG OR AB=EEG) OR (TI=electroencephalography OR AB=electroencephalography) OR ALL=electroencephalography OR (TI=MEG OR AB=MEG) OR (TI=magnetoencephalogram OR AB=magnetoencephalogram) OR (TI="brain imaging" OR AB="brain imaging"))
**#3**	((TI=graph OR AB=graph) OR (TI="network propert*" OR AB="network propert*") OR (TI="network characteristic*" OR AB="network characteristic*") OR (TI="network analys*" OR AB="network analys*") OR (TI="network theoretical analysis" OR AB="network theoretical analysis") OR (TI="global network" OR AB="global network") OR (TI="characteristic path length" OR AB="characteristic path length") OR (TI="global efficiency" OR AB="global efficiency") OR (TI="clustering coefficient" OR AB="clustering coefficient") OR (TI=transitivity OR AB=transitivity) OR (TI="local efficiency" OR AB="local efficiency") OR (TI=modularity OR AB=modularity) OR (TI="average degree" OR AB="average degree") OR (TI="average strength" OR AB="average strength") OR (TI="average eccentricity" OR AB="average eccentricity") OR (TI="small world*" OR AB="small world*") OR (TI="rich club*" OR AB="rich club*"))
**#4**	#1 AND #2 AND #3

**Initial search (Database start – 2024-04-29):**	698 Hits
**Second search (2024-04-29 – 2025-02-20):**	99 Hits
**Overall:**	**797 Hits**

Table 1.4 CINAHL via EBSCO

Search	Query
**#1**	((MH pain+) OR (TI pain* OR AB pain*) OR (TI "chronic pain" OR AB "chronic pain") OR (TI ache OR AB ache) OR (TI headache OR AB headache) OR (TI migraine OR AB migraine) OR (TI polyneuropathy OR AB polyneuropathy) OR (TI myalgia OR AB myalgia) OR (TI fibromyalgia OR AB fibromyalgia) OR (TI WAD OR AB WAD) OR (TI "whiplash associated disorder" OR AB "whiplash associated disorder") OR (TI "temporomandibular disorder" OR AB "temporomandibular disorder") OR (TI osteoarthritis OR AB osteoarthritis) OR (TI arthritis OR AB arthritis) OR (TI arthralgia OR AB arthralgia) OR (TI neuralgia OR AB neuralgia) OR (TI neuropathy OR AB neuropathy) OR (TI radiculopathy OR AB radiculopathy) OR (TI stenosis OR AB stenosis) OR (TI spondylolisthesis OR AB spondylolisthesis) OR (TI spondylosis OR AB spondylosis) OR (TI "disc hernia" OR AB "disc hernia") OR (TI "disc prolapse" OR AB "disc prolapse") OR (TI sciatica OR AB sciatica) OR (TI lumbago OR AB lumbago) OR (TI scoliosis OR AB scoliosis) OR (TI "sulcus ulnaris syndrome" OR AB "sulcus ulnaris syndrome") OR (TI "tunnel syndrome" OR AB "tunnel syndrome") OR (TI "frozen shoulder" OR AB "frozen shoulder") OR (TI "adhesive capsulitis" OR AB "adhesive capsulitis") OR (TI "temporomandibular joint disorder" OR AB "temporomandibular joint disorder") OR (TI "burning mouth syndrome" OR AB "burning mouth syndrome") OR (TI "spinal cord injur*" OR AB "spinal cord injur*") OR (TI CRPS OR AB CRPS) OR (TI "irritable bowel syndrome" OR AB "irritable bowel syndrome") OR (TI IBS OR AB IBS) OR (TI "inflammatory bowel disease" OR AB "inflammatory bowel disease") OR (TI IBD OR AB IBD) OR (TI crohn OR AB crohn) OR (TI colitis OR AB colitis) OR (TI cystitis OR AB cystitis) OR (TI endometriosis OR AB endometriosis) OR (TI prostatitis OR AB prostatitis) OR (TI pancreatitis OR AB pancreatitis) OR (TI angina OR AB angina) OR (TI "herpes zoster" OR AB "herpes zoster") OR (TI shingles OR AB shingles) OR (TI dyspepsia OR AB dyspepsia) OR (TI adenomyosis OR AB adenomyosis) OR (TI "pelvic congestion syndrome" OR AB "pelvic congestion syndrome") OR (TI vulvodynia OR AB vulvodynia) OR (TI "gastroesophageal reflux disease" OR AB "gastroesophageal reflux disease") OR (TI GERD OR AB GERD) OR (TI "peripheral artery diseas*" OR AB "peripheral artery diseas*") OR (TI PAD OR AB PAD) OR (TI burn* OR AB burn*))

**#2**	((MH connectome+) OR (TI connectome OR AB connectome) OR (TI connectivity OR AB connectivity) OR (TI brain OR AB brain) OR (TI topolog* OR AB topolog*) OR (TI "functional network*" OR AB "functional network*") OR (TI "functional architect*" OR AB "functional architect*") OR (TI "functional organi*" OR AB "functional organi*") OR (TI "structural network*" OR AB "structural network*") OR (TI "structural architect*" OR AB "structural architect*") OR (TI "structural organi*" OR AB "structural organi*") OR (TI "network architect*" OR AB "network architect*") OR (TI "network organi*" OR AB "network organi*") OR (MH "magnetic resonance imaging+") OR (TI MRI OR AB MRI) OR (TI fMRI OR AB fMRI) OR (TI "functional MRI" OR AB "functional MRI") OR (TI rs-fMRI OR AB rs-fMRI) OR (TI rsfmri OR AB rsfmri) OR (TI DTI OR AB DTI) OR (TI DKI OR AB DKI) OR (TI "diffusion tensor imaging" OR AB "diffusion tensor imaging") OR (TI "diffusion kurtosis imaging" OR AB "diffusion kurtosis imaging") OR (TI tractography OR AB tractography) OR (TI EEG OR AB EEG) OR (TI electroencephalography OR AB electroencephalography) OR (MH electroencephalography+) OR (TI MEG OR AB MEG) OR (TI magnetoencephalogram OR AB magnetoencephalogram) OR (TI "brain imaging" OR AB "brain imaging"))
**#3**	((TI graph OR AB graph) OR (TI "network propert*" OR AB "network propert*") OR (TI "network characteristic*" OR AB "network characteristic*") OR (TI "network analys*" OR AB "network analys*") OR (TI "network theoretical analysis" OR AB "network theoretical analysis") OR (TI "global network" OR AB "global network") OR (TI "characteristic path length" OR AB "characteristic path length") OR (TI "global efficiency" OR AB "global efficiency") OR (TI "clustering coefficient" OR AB "clustering coefficient") OR (TI transitivity OR AB transitivity) OR (TI "local efficiency" OR AB "local efficiency") OR (TI modularity OR AB modularity) OR (TI "average degree" OR AB "average degree") OR (TI "average strength" OR AB "average strength") OR (TI "average eccentricity" OR AB "average eccentricity") OR (TI "small world*" OR AB "small world*") OR (TI "rich club*" OR AB "rich club*"))
**#4**	#1 AND #2 AND #3
**Initial search (Database start – 2024-04-29):**	109 Hits
**Second search (2024-04-29 – 2025-02-20):**	10 Hits
**Overall:**	**119 Hits**

Table 1.5 PSYCINFO via EBSCO

Search	Query
**#1**	((MH pain+) OR (TI pain* OR AB pain*) OR (TI "chronic pain" OR AB "chronic pain") OR (TI ache OR AB ache) OR (TI headache OR AB headache) OR (TI migraine OR AB migraine) OR (TI polyneuropathy OR AB polyneuropathy) OR (TI myalgia OR AB myalgia) OR (TI fibromyalgia OR AB fibromyalgia) OR (TI WAD OR AB WAD) OR (TI "whiplash associated disorder" OR AB "whiplash associated disorder") OR (TI "temporomandibular disorder" OR AB "temporomandibular disorder") OR (TI osteoarthritis OR AB osteoarthritis) OR (TI arthritis OR AB arthritis) OR (TI arthralgia OR AB arthralgia) OR (TI neuralgia OR AB neuralgia) OR (TI neuropathy OR AB neuropathy) OR (TI radiculopathy OR AB radiculopathy) OR (TI stenosis OR AB stenosis) OR (TI spondylolisthesis OR AB spondylolisthesis) OR (TI spondylosis OR AB spondylosis) OR (TI "disc hernia" OR AB "disc hernia") OR (TI "disc prolapse" OR AB "disc prolapse") OR (TI sciatica OR AB sciatica) OR (TI lumbago OR AB lumbago) OR (TI scoliosis OR AB scoliosis) OR (TI "sulcus ulnaris syndrome" OR AB "sulcus ulnaris syndrome") OR (TI "tunnel syndrome" OR AB "tunnel syndrome") OR (TI "frozen shoulder" OR AB "frozen shoulder") OR (TI "adhesive capsulitis" OR AB "adhesive capsulitis") OR (TI "temporomandibular joint disorder" OR AB "temporomandibular joint disorder") OR (TI "burning mouth syndrome" OR AB "burning mouth syndrome") OR (TI "spinal cord injur*" OR AB "spinal cord injur*") OR (TI CRPS OR AB CRPS) OR (TI "irritable bowel syndrome" OR AB "irritable bowel syndrome") OR (TI IBS OR AB IBS) OR (TI "inflammatory bowel disease" OR AB "inflammatory bowel disease") OR (TI IBD OR AB IBD) OR (TI crohn OR AB crohn) OR (TI colitis OR AB colitis) OR (TI cystitis OR AB cystitis) OR (TI endometriosis OR AB endometriosis) OR (TI prostatitis OR AB prostatitis) OR (TI pancreatitis OR AB pancreatitis) OR (TI angina OR AB angina) OR (TI "herpes zoster" OR AB "herpes zoster") OR (TI shingles OR AB shingles) OR (TI dyspepsia OR AB dyspepsia) OR (TI adenomyosis OR AB adenomyosis) OR (TI "pelvic congestion syndrome" OR AB "pelvic congestion syndrome") OR (TI vulvodynia OR AB vulvodynia) OR (TI "gastroesophageal reflux disease" OR AB "gastroesophageal reflux disease") OR (TI GERD

	OR AB GERD) OR (TI "peripheral artery diseas*" OR AB "peripheral artery diseas*") OR (TI PAD OR AB PAD) OR (TI burn* OR AB burn*))
**#2**	((MH connectome+) OR (TI connectome OR AB connectome) OR (TI connectivity OR AB connectivity) OR (TI brain OR AB brain) OR (TI topolog* OR AB topolog*) OR (TI "functional network*" OR AB "functional network*") OR (TI "functional architect*" OR AB "functional architect*") OR (TI "functional organi*" OR AB "functional organi*") OR (TI "structural network*" OR AB "structural network*") OR (TI "structural architect*" OR AB "structural architect*") OR (TI "structural organi*" OR AB "structural organi*") OR (TI "network architect*" OR AB "network architect*") OR (TI "network organi*" OR AB "network organi*") OR (MH "magnetic resonance imaging+") OR (TI MRI OR AB MRI) OR (TI fMRI OR AB fMRI) OR (TI "functional MRI" OR AB "functional MRI") OR (TI rs-fMRI OR AB rs-fMRI) OR (TI rsfmri OR AB rsfmri) OR (TI DTI OR AB DTI) OR (TI DKI OR AB DKI) OR (TI "diffusion tensor imaging" OR AB "diffusion tensor imaging") OR (TI "diffusion kurtosis imaging" OR AB "diffusion kurtosis imaging") OR (TI tractography OR AB tractography) OR (TI EEG OR AB EEG) OR (TI electroencephalography OR AB electroencephalography) OR (MH electroencephalography+) OR (TI MEG OR AB MEG) OR (TI magnetoencephalogram OR AB magnetoencephalogram) OR (TI "brain imaging" OR AB "brain imaging"))
**#3**	((TI graph OR AB graph) OR (TI "network propert*" OR AB "network propert*") OR (TI "network characteristic*" OR AB "network characteristic*") OR (TI "network analys*" OR AB "network analys*") OR (TI "network theoretical analysis" OR AB "network theoretical analysis") OR (TI "global network" OR AB "global network") OR (TI "characteristic path length" OR AB "characteristic path length") OR (TI "global efficiency" OR AB "global efficiency") OR (TI "clustering coefficient" OR AB "clustering coefficient") OR (TI transitivity OR AB transitivity) OR (TI "local efficiency" OR AB "local efficiency") OR (TI modularity OR AB modularity) OR (TI "average degree" OR AB "average degree") OR (TI "average strength" OR AB "average strength") OR (TI "average eccentricity" OR AB "average eccentricity") OR (TI "small world*" OR AB "small world*") OR (TI "rich club*" OR AB "rich club*"))
**#4**	#1 AND #2 AND #3
**Initial search (Database start – 2024-04-29):**<br>**Second search (2024-04-29 – 2025-02-20):**<br>**Overall:**	128 Hits<br>12 Hits<br>**140 Hits**

2 Appendix 2 – Additional methods

2.1 Formulas used in data extraction and GRADE criteria

Table 2.1 Calculations used in data extraction in the review

**Used for**	**Calculation**
Standard Error to Standard Deviation (Cochrane Handbook)	$SE\sqrt{n}$
Median to Mean (Wan et al., 2014)	$\frac{Q1 + Md + Q3}{3}$ or $\frac{Min + 2 * Md + Max}{4}$
Minimum, Median, Maximum to SD (Wan et al., 2014)	$\frac{Max - Min}{2 * qnorm(\frac{n - 0.375}{n + 0.25}, 0,1)}$
1. Quartile, Median, 3. Quartile to SD (Wan et al., 2014)	$\frac{Q3 - Q1}{2 * qnorm(\frac{0.75 * n - 0.125}{n + 0.25}, 0,1)}$
Pooling Mean (Cochrane Handbook)	$\frac{N_1M_1 + N_2M_2}{N_1 + N_2}$
Pooling SD (Cochrane Handbook)	$\sqrt{\frac{(N_1 - 1)SD_1^2 + (N_2 - 1)SD_2^2 + \frac{N_1N_2}{N_1 + N_2}(M_1^2 + M_2^2 - 2M_1M_2)}{N_1 + N_2 - 1}}$
Pooling Numbers (Cochrane Handbook)	$N_1 + N_2$

Table 2.2 GRADE criteria

**GRADE Domain**	**Criteria used in the review**
Risk of Bias	• Downgraded by one level if estimate included studies with some concerns or at a high risk of bias.<br>• Downgraded by two levels if all studies informing the meta-analysis were high risk of bias.
Inconsistency	• Downgraded by one level if substantial heterogeneity was present ($I^2 > 50\%$ or prediction interval crossing zero).
Imprecision	• Downgraded by one level if the 95% confidence interval of the effect estimate includes no effect and the upper or lower limit crosses an effect (Hedge g) of 0.5
Publication Bias	• Downgraded by one level if evidence of asymmetry in funnel plots was indicated by the adapted Eggers regression test or by known studies excluded from meta-analysis due to poor outcome

	reporting. The publication bias assessment from the main analysis was used to GRADE the subgroup analysis if available.

2.2 Data extraction with 'WebPlotDigitizer'

- Take a screenshot of the plot
- Launch WebPlotDigitizer (v5) and upload the screenshot of the plot
- Select appropriate axe type:

Lineplots (➔ '2D XY Axes')

- Click to define 4 points (X1, X2, Y1, Y2) on the X- and Y-axis of the plot
- On the right side: Define the values of the points & hit 'Calibrate'
- On the left side: Create a dataset (CAVE: Naming = *metric_group_raw* or *metric_group_subgroup_raw)*
- Add points for each mean value and upper SD value

Barplots (➔ 'Bar Chart')

- Add points for each mean and upper SD value
- Name the points according to group (e.g.: *hc*, *cp*; if subgroups extracted use *group_subgroup*)

Boxplots (➔ 'Bar Chart')

- Add points for each 25% quartile, median and 75% quartile
- Name the points according to group

- Download the data for each dataset using 'View data' (left panel) and save it in a folder
  - Run the appropriate R-script to compute the mean and SD for the variable of interest ('WebPlotDigitizer_lineplot.R', 'WebPlotDigitizer_barplot.R', 'WebPlotDigitizer_barplot.R'; Download here: https://osf.io/y27qc/)
  - CAVE: Manually specify the 'USER INPUT' in the script.

2.3 Newcastle-Ottawa Scale – Coding manual adapted for the 'NET-PAIN' review

SELECTION

1) Is the case definition adequate?

a) Requires some independent validation (e.g. >1 person/record/time/process to extract information or reference to primary record sources such as x-rays or medical/hospital records) *
b) Record linkage (e.g. ICD codes in the database) or self-report with no reference to the primary record
c) No description

2) Representativeness of cases

a) All eligible cases with the outcome of interest over a defined period of time, all cases in a defined catchment area, all cases in a defined hospital or clinic, group of hospitals, health maintenance organisation, or an appropriate sample of those cases (e.g. random sample) *
b) Not satisfying requirements in part (a), or not stated.

3) Selection of controls

This item assesses whether the control series used in the study is derived from the same population as the cases and essentially would have been cases had the outcome been present.

a) Community controls (i.e. same community as cases and would be cases if had outcome) *
b) Hospital controls, within same community as cases (i.e. not another city) but derived from a hospitalised population
c) No description

4) Definition of Controls

a) If cases are first occurrence of outcome, then it must explicitly state that controls have no history of this outcome. If cases have new (not necessarily first) occurrence of outcome, then controls with previous occurrences of outcome of interest should not be excluded. *
b) No mention of history of outcome

COMPARABILITY

A maximum of 2 stars can be allotted in this category. Either cases and controls must be matched in the design and/or confounders must be adjusted for in the analysis. Statements of no differences between groups or that differences were not statistically significant are not sufficient for establishing comparability. Note: If the group difference for the outcome is adjusted for the confounders listed, then the groups will be considered to be comparable on each variable used in the adjustment.

*a) Study controls for age **
*b) Study controls for age and sex ***
c) Study does not control for any variable

EXPOSURE (here: assessment of brain network organization)

1) Ascertainment of exposure

a) Appropriate imaging technique, network calculation and reporting of methods *
b) No description or poor reporting of methods

2) Same method of ascertainment for cases and controls

a) Yes *
b) No

3) Non-response rate / dropouts

Refers both to non-responders and exclusion of datasets due to various reasons (e.g. imaging quality)

a) Same rate for both groups *
b) Non-respondents / dropouts described
c) Rate different and no designation

<u>Maximum of 9 stars can be allocated for a study.</u>

3 Appendix 3 – Studies excluded at the full-text phase with reasons

Table 3.1 Studies excluded at full-text screening with reason (n=84)

Author	Year	Title	Reason
Alizadeh et al.	2021	Graph theoretical structural connectome analysis of the brain in patients with chronic spinal cord injury: preliminary investigation	IS NOT chronic pain
Amarasinghe et al.	2024	Graph Theory Structural brain network topology in migraine vs. healthy subjects: A graph theory study	IS structural covariance network
Bai et al.	2009	Detection of dynamic brain networks modulated by acupuncture using a graph theory model	IS NOT chronic pain
Bao et al.	2023	Altered cortical thickness and structural covariance networks in upper limb amputees: A graph theoretical analysis	IS structural covariance network
Cao et al.	2024	Right-to-left shunt-associated brain functional changes in migraine: Evidences from a resting-state FMRI study.	NO healthy control group
Case et al.	2019	Graph theory analysis reveals how sickle cell disease impacts neural networks of patients with more severe disease	IS including subjects <18y
Chen et al.	2021	Calcarine as a bridge between brain function and structure in irritable bowel syndrome: A multiplex network analysis	NO outcome measure of interest
Chen et al.	2023	Graph theory analysis reveals an assortative pain network vulnerable to attacks	IS animal study
Chen et al.	2024	Abnormal Topological Organization of Human Brain Connectome in Primary Dysmenorrhea Patients Using Graph Theoretical Analysis	IS NOT chronic pain
Dai et al.	2021	Altered local and distant functional connectivity density in chronic migraine: a resting-state functional MRI study	NO outcome measure of interest
Dai et al.	2023	Abnormal Thalamo–Cortical Interactions in Overlapping Communities of Migraine: An Edge Functional Connectivity Study	NO outcome measure of interest
Davis et al.	2016	Identifying brain nociceptive information transmission in patients with chronic somatic pain	NO outcome measure of interest
Deng et al.	2023	Exploring neural activity in inflammatory bowel diseases using functional connectivity and DKI-fMRI fusion	IS NOT chronic pain
De Souza et al.	2020	Altered structural brain network topology in chronic migraine	IS structural covariance network
De Tommaso et al.	2015	Functional Connectivity of EEG Signals Under Laser Stimulation in Migraine	IS task-based imaging
Engels et al.	2018	Clinical pain and functional network topology in Parkinson's disease: a resting-state fMRI study	NO healthy control group
Gu et al.	2024	Altered cortical thickness and structural covariance networks in chronic low back pain	IS structural covariance network
Guo e al.	2019	Alterations in Brain Structural Connectivity After Unilateral Upper-Limb Amputation	IS NOT chronic pain
Gupta et al.	2019	Impact of early adverse life events and sex on functional brain networks in patients with urological chronic pelvic pain syndrome (UCPPS): A MAPP Research Network study	NO outcome measure of interest
Ha et al.	2019	Alterations of structural connectivity in episodic cluster headache: A graph theoretical analysis	IS structural covariance network
Hasan et al.	2024	Brain Networks With Modified Connectivity in Patients With Neuropathic Pain and Spinal Cord Injury	IS task-based imaging
Jiang et al.	2024	Resting state functional connectivity of the pain matrix and default mode network in irritable bowel syndrome: a graph theoretical analysis	NO outcome measure of interest
Kano et al.	2020	Resting state functional connectivity of the pain matrix and default mode network in irritable bowel syndrome: a graph theoretical analysis	IS NOT whole brain analysis
Kushal et al.	2017a	Large-Scale Network Analysis of Whole-Brain Resting-State Functional Connectivity in Spinal Cord Injury: A Comparative Study	IS NOT chronic pain
Kaushal et al.	2017b	Evaluation of Whole-Brain Resting-State Functional Connectivity in Spinal Cord Injury: A Large-Scale Network Analysis Using Network-Based Statistic	IS NOT chronic pain
Khodayari-Rostamabad et al.	2015	Disruption of Cortical Connectivity during Remifentanil Administration Is Associated with Cognitive Impairment but Not with Analgesia	IS NOT chronic pain
Kim et al.	2015	Fibromyalgia is characterized by altered frontal and cerebellar structural covariance brain networks	NO outcome measure of interest

Kim et al.	2020	Comparison Analysis between the Medication Efficacy of the Milnacipran and Functional Connectivity of Neural Networks in Fibromyalgia Patients	NO healthy control group
Kim et al.	2023	Multilayer network changes in patients with migraine	NO outcome measure of interest
Labus et al.	2019	Evidence for an association of gut microbial Clostridia with brain functional connectivity and gastrointestinal sensorimotor function in patients with irritable bowel syndrome, based on tripartite network analysis	NO outcome measure of interest
Lamichhane et al.	2021	Functional Disruptions of the Brain in Low Back Pain: A Potential Imaging Biomarker of Functional Disability	NO outcome measure of interest
Lan et al.	2022	Aberrant Modulations of Neurocognitive Network Dynamics in Migraine Comorbid With Tinnitus	NO outcome measure of interest
Larocca et al.	2023	Galcanezumab treatment changes visual related EEG connectivity patterns in migraine patients	IS task-based imaging
Lee et al.	2018	Unaltered intrinsic functional brain architecture in young women with primary dysmenorrhea	IS NOT chronic pain
Lee et al.	2019	Increased connectivity of pain matrix in chronic migraine: a resting-state functional MRI study	NO outcome measure of interest
Lee et al.	2022	Alterations of limbic structure volumes and limbic covariance network in patients with cluster headache	IS structural covariance network
Letzen et al.	2020	Altered mesocorticolimbic functional connectivity in chronic low back pain patients at rest and following sad mood induction	IS NOT whole brain analysis
Li et al.	2021	A Resting-state Functional Magnetic Resonance Imaging Study of Whole-brain Functional Connectivity of Voxel Levels in Patients With Irritable Bowel Syndrome With Depressive Symptoms	NO outcome measure of interest
Li et al.	2022	Temporal Grading Index of Functional Network Topology Predicts Pain Perception of Patients With Chronic Back Pain	IS NOT comparative design
Li et al.	2023a	Greater functional connectivity between the ventral frontal cortex and occipital cortex in herpes zoster patients than post-herpetic neuralgia patients	IS NOT chronic pain
Li et al.	2023b	EEG assessment of brain dysfunction for patients with chronic primary pain and depression under auditory oddball task	IS task-based imaging
Li et al.	2023c	Menstrually-related migraine shapes the structural similarity network integration of brain	IS structural covariance network
Li et al.	2025a	Abnormal Alterations of the White Matter Structural Network in Patients with Herpes Zoster and Postherpetic Neuralgia	IS NOT chronic pain
Li et al.	2025b	Abnormal alterations in structure-function coupling at the modular level in patients with postherpetic neuralgia	IS NOT chronic pain
Liu et al.	2015	Disrupted resting-state functional connectivity and its changing trend in migraine suffers	IS NOT comparative design
Liu et al.	2018	Altered topological patterns of brain functional networks in Crohn's disease	IS NOT chronic pain
Liu et al.	2023	Alterations of degree centrality and functional connectivity in classic trigeminal neuralgia	NO outcome measure of interest
Ma et al.	2020	Brain Functional Interaction of Acupuncture Effects in Diarrhea-Dominant Irritable Bowel Syndrome	NO outcome measure of interest
Meier et al.	2020	Network Analysis of Induced Neural Plasticity Post-Acceptance and Commitment Therapy for Chronic Pain	NO healthy control group
Michels et al.	2011	Structural brain network characteristics in patients with episodic and chronic migraine	IS structural covariance network
Min et al.	2015	Change of Brain Functional Connectivity in Patients With Spinal Cord Injury: Graph Theory Based Approach	IS NOT chronic pain
Nan et al.	2015	Topological Alterations of the Intrinsic Brain Network in Patients with Functional Dyspepsia	IS NOT chronic pain
Pahapill et al.	2024	Distinct Functional Connectivity Patterns for Intermittent Vs Constant Neuropathic Pain Phenotypes in Persistent Spinal Pain Syndrome Type 2 Patients	NO outcome measure of interest
Park et al.	2023	Effects of Alterations in Resting-State Neural Networks on the Severity of Neuropathic Pain after Spinal Cord Injury	NO outcome measure of interest
Qi et al.	2016	Topological Reorganization of the Default Mode Network in Irritable Bowel Syndrome	IS NOT whole brain analysis
Ren et al.	2019	Abnormal functional connectivity under somatosensory stimulation in migraine: a multi-frequency magnetoencephalography study	IS task-based imaging

Ren et al.	2022	Altered effective connectivity in migraine patients during emotional stimuli: a multi-frequency magnetoencephalography study	IS task-based imaging
Roine et al.	2022	Structural Brain Connectivity Correlates with Outcome in Mild Traumatic Brain Injury	IS NOT chronic pain
Santana et al.	2023	Altered Dynamic Brain Connectivity in Individuals With Sickle Cell Disease and Chronic Pain Secondary to Hip Osteonecrosis	NO outcome measure of interest
Tsai et al.	2019	Modular organization of brain resting state networks in patients with classical trigeminal neuralgia	IS NOT whole brain analysis
Tu et al.	2019	Abnormal medial prefrontal cortex functional connectivity and its association with clinical symptoms in chronic low back pain	NO outcome measure of interest
Turkiewicz et al.	2021	Altered brain structural connectivity in patients with longstanding gut inflammation is correlated with psychological symptoms and disease duration	NO outcome measure of interest
Vuckovic et al.	2018	Central neuropathic pain in paraplegia alters movement related potentials	IS task-based imaging
Wang et al.	2021	Altered Topological Properties of Grey Matter Structural Covariance Networks in Complete Thoracic Spinal Cord Injury Patients: A Graph Theoretical Network Analysis	IS structural covariance network
Wu et al.	2021	Dynamic network topological properties for classifying primary dysmenorrhoea in the pain-free phase	NO outcome measure of interest
Wu et al.	2023	Disrupted Brain Functional Status in Patients with Reversible Cerebral Vasoconstriction Syndrome	IS NOT chronic pain
Wu et al.	2024a	Higher-order sensorimotor circuit of the whole-brain functional network involved in pruritus regulation in atopic dermatitis	IS NOT chronic pain
Wu et al.	2024b	A study of brain functional networks in patients with inflammatory bowel disease based on graph theory	IS NOT English/German
Xin et al.	2024	Cognition and motion dysfunction-associated brain functional network disruption in diabetic peripheral neuropathy	IS NOT chronic pain
Xing et al.	2021	Abnormal Brain Connectivity in Carpal Tunnel Syndrome Assessed by Graph Theory	IS NOT chronic pain
Xu et al.	2021	Distributed Functional Connectome of White Matter in Patients With Functional Dyspepsia	IS NOT chronic pain
Xu et al.	2024	Abnormal longitudinal changes of structural covariance networks of cortical thickness in mild traumatic brain injury with posttraumatic headache	IS structural covariance network
Yang et al.	2020	Aberrant Brain Network Integration and Segregation in Diabetic Peripheral Neuropathy Revealed by Structural Connectomics	IS structural covariance network
Yu et al.	2023	Altered intrinsic functional brain architecture in patients with functional constipation: a surface-based network study	IS NOT chronic pain
Zhang et al.	2021	Altered brain functional network dynamics in classic trigeminal neuralgia: a resting-state functional magnetic resonance imaging study	NO outcome measure of interest
Zhang et al.	2022c	Aberrant resting-state functional connectivity and topological properties of the subcortical network in functional dyspepsia patients	IS NOT chronic pain
Zhang et al.	2022d	Functional and structural alterations in the pain-related circuit in major depressive disorder induced by electroconvulsive therapy	NO outcome measure of interest
Zheng et al.	2020	Pain-Evoked Reorganization in Functional Brain Networks	IS NOT chronic pain
Zhou et al.	2024	Altered Static and Dynamic Brain Functional Topological Organization in Patients With Dysthyroid Optic Neuropathy	IS NOT chronic pain
Zhu et al.	2020	Altered brain network centrality in patients with trigeminal neuralgia: a resting-state fMRI study	NO outcome measure of interest
Mano et al.	2018	Classification and characterisation of brain network changes in chronic back pain: A multicenter study	NO outcome measure of interest
Smith et al.	2022	Topology of pain networks in patients with temporomandibular disorder and pain-free controls with and without concurrent experimental pain: A pilot study	IS task-based imaging
Yang et al.	2023	Reorganization of brain networks in patients with temporal lobe epilepsy and comorbid headache	IS NOT chronic pain

4 Appendix 4 – Study characteristics of included studies

Table 4.1: Study characteristics of studies investigating functional brain topology

	**Population characteristics**							**Imaging characteristics**			**Network computation characteristics**			
**Study**	**Group**	**Pain type (ICD-11)**	**Group size (n)**	**Sex (♂)**	**Age (years)**	**Pain duration (months)**	**Pain intensity (NRS)**	**Modality**	**Field strength (T) and/or sampling frequency (Hz)**	**Voxel dimensions (mm) or number of channels**	**Parcellation (n)**	**Edge type (Sparsity, %)**	**Soft-ware**	**Edge metric**
Balenzuela et al., 2010	Chronic back pain<br>Control	CPP<br>-	12<br>12	NR<br>NR	51.2 ± 11.2<br>40.2 ± 12.7	NR<br>-	NR<br>-	rs-fMRI	3 T<br>0.01 – 0.1 Hz	non-iso<br>(3.1×3.1×3.3)	AAL<br>(90)	W<br>(60 – 90)	NR	Partial correlation
Barroso et al., 2021	Knee OA<br>Control	CSP<br>-	46<br>35	35%<br>43%	65.3 ± 7.41<br>59.5 ± 7.91	81.6 ± 65.4<br>-	6.5 ± 1.4<br>-	rs-fMRI	3 T<br>0.008 – 0.1 Hz	non-iso<br>(3.4×3.4×3)	Power2011<br>(256)	B<br>(2 – 10)	BCT	Full correlation
De Pauw et al., 2020	Idiopathic Neck pain<br>WAD<br>Control	CPP<br>CSP<br>-	39<br>37<br>35	0%<br>0%<br>0%	37.1 ± 12.2<br>37.6 ± 12<br>30.4 ± 12.3	85.2 ± 82.1<br>88.9 ± 89.4<br>-	2.9 ± 2.2<br>5.8 ± 2.2<br>-	rs-fMRI	3 T<br>0.009 – 0.08 Hz	iso<br>(3×3×3)	DKA<br>(84)	B<br>(30 – 70)	BCT	Full correlation
Duan et al., 2021	FCwAD<br>FCwoAD<br>Control	CSP<br>CSP<br>-	41<br>42<br>43	24%<br>38%<br>42%	38.6 ± 11.5<br>42.8 ± 13.5<br>38.1 ± 13.7	NR<br>NR<br>-	3.5 ± 3<br>2.6 ± 2.4<br>-	rs-fMRI	1.5 T<br>0.01 – 0.08 Hz	iso<br>(4×4×4)	Brainnetome<br>(246)	B<br>(10 – 30)	GRETNA	Full correlation
Fauchon et al., 2021	sCLBP (♂)<br>Control (♂)<br>sCLBP (♀)<br>Control (♀)	CSP<br>-<br>CSP<br>-	45<br>45<br>20<br>20	100%<br>100%<br>0%<br>0%	34.5 ± 10.1<br>33.8 ± 10<br>32.2 ± 10.3<br>32.3 ± 10.3	176.4 ± 97.2<br>-<br>182.4 ± 129.6<br>-	NR<br>-<br>NR<br>-	rs-fMRI	3 T<br>0.01 - NR Hz	non-iso<br>(3.1×3.1×4)	Glasser<br>(360)	W<br>(5)	Radatools	Full correlation
Huang et al., 2019	sCLBP<br>Control	CSP<br>-	68<br>68	63%<br>60%	44 ± 12.4<br>43.7 ± 14	93.3 ± 9.69<br>-	5 ± 1.5<br>-	rs-fMRI	3 T<br>0.008 – 0.1 Hz	non-iso<br>(3.4×3.4×3.5)	Power2011<br>(256)	B<br>(2 – 10)	BCT	Full correlation
Kang et al., 2024	Knee OA<br>Control	CSP<br>-	46<br>31	37%<br>32%	70.3 ± 5.5<br>68.4 ± 3.8	108 ± 37.2<br>-	6.5 ± 0.8<br>-	rs-fMRI	1.5 T<br>0.01 – 0.08 Hz	iso<br>(3.5×3.5×3.5)	AAL<br>(90)	B<br>(15 – 45)	GRETNA	Full correlation
Kaplan et al., 2019	Fibromyalgia<br>Control	CPP<br>-	40<br>46	0%<br>0%	39 ± 11<br>38.8 ± 12.2	NR<br>-	4.8 ± 2.24<br>-	rs-fMRI	3 T<br>0.008 – 0.09 Hz	non-iso<br>(3.1×3.1×4)	Power2011<br>(264)	B<br>(5 – 40)	BCT	Full correlation
Larkin et al., 2021	Fibromyalgia<br>Control	CPP<br>-	38<br>17	0%<br>0%	41 ± 12.1<br>43.8 ± 10.4	NR<br>-	5.4 ± 2<br>-	rs-fMRI	3 T<br>0.01 - 0.1 Hz	non-iso<br>(3.1×3.1×3.3)	Power2011<br>(264)	W<br>(absolute: 0.5)	BCT	Full correlation
Lin et al., 2022	Knee OA<br>Control	CSP<br>-	52<br>22	23%<br>86%	70.3 ± 4.3<br>70.6 ± 4.6	NR<br>-	5.7 ± 2.3<br>-	rs-fMRI	3 T<br>NR	non-iso<br>(3.7×3.7×4)	AAL<br>(116)	NR<br>(5 – 50)	GRETNA	Full correlation
Liu et al., 2011	Episodic Migraine (♂)<br>Control (♂)<br>Episodic Migraine (♀)<br>Control (♀)	CPP<br>-<br>CPP<br>-	18<br>18<br>20<br>20	100%<br>100%<br>0%<br>0%	33 ± 7.9<br>33.3 ± 2.1<br>32.3 ± 8.7<br>31.1 ± 9.3	97.2 ± 27.6<br>-<br>130.8 ± 88.8<br>-	5.2 ± 1.2<br>-<br>5.4 ± 1.7<br>-	rs-fMRI	3 T<br>0.01 – 0.08 Hz	iso<br>(2×2×2)	AAL<br>(90)	B<br>(15 – 25)	NR	Full correlation
Liu et al., 2012	Episodic Migraine<br>Control	CPP<br>-	43<br>43	0%<br>0%	32.6 ± 11.1<br>33.4 ± 10.2	174 ± 81.6<br>-	5.5 ± 1.6<br>-	rs-fMRI	3 T<br>0.01 – 0.1 Hz	non-iso<br>(3.7×3.7×5)	AAL<br>(90)	B<br>(15 – 30)	NR	Full correlation
Liu et al., 2018	sCLBP<br>Control	CSP<br>-	20<br>17	NR<br>NR	73.2 ± NR<br>70.1 ± NR	NR<br>-	6.6 ± NR<br>-	rs-fMRI	3 T<br>0.01 – 0.08 Hz	non-iso<br>(3×3×4)	AAL<br>(116)	NR<br>(20)	GRETNA	Full correlation
Mansour et al., 2016	CRPS<br>OA<br>Back Pain<br>Control	CPP<br>CSP<br>CPP<br>-	22<br>20<br>25<br>75	18%<br>60%<br>52%<br>41%	42.4 ± 12.1<br>54.2 ± 6.1<br>47.6 ± 9.2<br>44.2 ± 11.1	36.8 ± 48.6<br>122.2 ± 117.9<br>189.1 ± 141.6<br>-	5.2 ± 2.1<br>7.9 ± 2.3<br>6.8 ± 1.9<br>-	rs-fMRI	3 T<br>NR	iso<br>(6×6×6)	Voxel-wise<br>(5828)	NR<br>(1 – 5)<br>(NMI: 10)	BCT	Full correlation
Mao et al., 2024	Knee OA<br>Control	CSP<br>-	27<br>27	15%<br>15%	55.1 ± 8.9<br>50.8 ± 8	88.8 ± 109.2<br>-	4.6 ± 1.8<br>-	rs-fMRI	3 T<br>0.01 – 0.08 Hz	non-iso<br>(4×4×3)	HOA-aggregated<br>(8)	NR<br>(5 – 50)	GRETNA	Full correlation
Nieboer et al., 2020	Migraine<br>Control	CPP<br>-	24<br>24	21%<br>17%	42 ± 0.61<br>42 ± 0.7	NR<br>-	NR<br>-	rs-MEG	0.5 – 4 Hz<br>4 – 8 Hz<br>8 – 10 Hz<br>10 – 13 Hz	151 channels	AAL<br>(78)	W<br>(Minimum spanning tree)	NR	Phase Lag Index

									13 – 30 Hz					
Qiu et al., 2023	NDPH<br>Control	CPP<br>-	35<br>40	49%<br>40%	40.6 ± 15.7<br>38 ± 13	39 ± 58<br>-	5.7 ± 3.1<br>-	rs-MEG	1 – 4 Hz<br>4 – 8 Hz<br>8 – 13 Hz<br>13 – 30 Hz<br>30 – 80 Hz	306 channels	DKA<br>(68)	NR<br>(5 – 39)	GRETNA	Envelope correlations of ortho-gonalized activity
Shi et al., 2019	Migraine<br>Control	CPP<br>-	24<br>22	42%<br>45%	45.6 ± NR<br>43.5 ± NR	NR<br>-	NR<br>-	rs-fMRI	3 T<br>0.01 – 0.1	iso<br>(4×4×4)	AAL<br>(90)	B<br>(10 – 30)	NR	Partial correlation
Shi et al., 2020	Episodic/Chronic Migraine<br>Control	CPP<br>-	48<br>49	NR<br>NR	NR<br>NR	NR<br>-	NR<br>-	rs-fMRI	3 T<br>NR	iso<br>(4×4×4)	ICA-based<br>(9)	B<br>(none)	NR	Full correlation
Ta Dinh et al., 2019	(Back pain; Widespread pain; Joint pain; unspecific neuropathic; Postherpetic neuralgia, PNP pain)<br>Control	Mixed<br>-	101<br>84	32%<br>35%	58.2 ± 13.5<br>57.8 ± 14.6	121.9 ± 114.3<br>-	5.7 ± 1.6<br>-	rs-EEG	4 – 8 Hz<br>8 – 13 Hz<br>14 – 30 Hz<br>60 – 100 Hz	64 channels	Voxel-based<br>(2020)	B<br>(10)	BCT	Phase Locking Value
Tu et al., 2019	Episodic migraine<br>Control	CPP<br>-	89<br>70	25%<br>26%	22 ± 2.3<br>22.2 ± 1	68 ± 34.7<br>-	5.6 ± 1.1<br>-	rs-fMRI	3 T<br>NR - 0.15 Hz	non-iso<br>(3.7×3.7×5)	ICA-based<br>(52)	B<br>(10 – 35)	GRETNA	Full correlation
Wang et al., 2022	IBS<br>Control	CPP<br>-	74<br>74	47%<br>47%	34 ± 8.2<br>34.3 ± 8.2	228 ± 125.5<br>-	NR<br>-	rs-fMRI	3 T<br>0.01 – 0.08 Hz	non-iso<br>(3.4×3.4×4)	Schaefer<br>(400)	W<br>(10)	BCT	Full correlation
Wang et al., 2024	CTN<br>Control	CSP<br>-	33<br>21	30%<br>NR	62.4 ± 10.6<br>-	67.1 ± 78.8<br>-	8.2 ± 1.1<br>-	rs-fMRI	3 T<br>0.01 – 0.08 Hz	non-iso<br>(3.7×3.7×4)	AAL<br>(90)	B<br>(5 – 40)	GRETNA	Full correlation
Wu et al., 2016	Episodic migraine<br>Control	CPP<br>-	23<br>23	35%<br>35%	30 ± 6.6<br>29.8 ± 6.9	NR<br>-	6 ± 1.5<br>-	rs-MEG	0.1 – 1 Hz<br>1 – 4 Hz<br>4 – 8 Hz<br>8 – 12 Hz<br>12 – 30 Hz<br>30 – 80 Hz<br>80 – 250 Hz	275 channels	Channel-based<br>(275)	NR<br>(NR)	NR	Coherence
Yang et al., 2023	nsCLBP<br>Control	CPP<br>-	33<br>34	55%<br>59%	49.4 ± 8.7<br>50.5 ± 7.8	177.8 ± 129.6<br>-	6.6 ± 1.6<br>-	rs-fMRI	3 T<br>0.01 – 0.08 Hz	NR	AAL<br>(90)	B<br>(1 – 34)	GRETNA	Full correlation
Yang et al., 2024	IC-SCI with NP<br>C-SCI with NP<br>Control	CSP<br>CSP<br>-	15<br>20<br>32	60%<br>75%<br>81%	42.5 ± 18.1<br>39.3 ± 16.3<br>44.1 ± 12.2	85 ± 109.1<br>87.5 ± 84.1<br>-	5.4 ± 3.7<br>6.6 ± 3.5<br>-	rs-fMRI	3 T<br>0.01 – 0.08 Hz	non-iso<br>(3.4×3.4×3)	AAL<br>(90)	NR<br>(1 – 34)	GRETNA	Full correlation
Yang et al., 2025	IBS<br>Control	CPP<br>-	21<br>20	62%<br>55%	35.5 ± 7.4<br>36.2 ± 10.1	14.5 ± 4.7<br>-	NR<br>-	rs-fMRI	3 T<br>0.01 – 0.08 Hz	non-iso<br>(3×3×4)	AAL<br>(90)	B<br>(1 – 50)	GRETNA/ BCT	Full correlation
Zhang et al., 2014	PHN<br>Control	CSP<br>-	16<br>16	50%<br>50%	68.1 ± 8.2<br>68.6 ± NR	9.4 ± 6.5<br>-	7.6 ± 0.9<br>-	rs-fMRI	3 T<br>0.01 – 0.08 Hz	non-iso<br>(3.6×3.6×8)	AAL<br>(90)	B<br>(1 – 100)	NR	Full correlation
Zhang et al., 2017	Episodic migraine<br>Control	CPP<br>-	29<br>29	0%<br>0%	40.1 ± 11.4<br>39.8 ± 11.2	112.8 ± 88.8<br>-	6.8 ± 2<br>-	rs-fMRI	3 T<br>0.01 – 0.1 Hz	iso<br>(3.5×3.5×3.5)	Brainnetome<br>(246)	B<br>(1 – 30)	GRETNA	Full correlation
Zhang et al., 2022a	CTN<br>Control	CSP<br>-	29<br>34	31%<br>35%	54.6 ± 10.8<br>55 ± 6.8	49.4 ± 39<br>-	7.9 ± 0.9<br>-	rs-fMRI	3 T<br>0.01 – 0.1 Hz	non-iso<br>(3×3×3.4)	Brainnetome<br>(246)	B<br>(NR)	GRETNA	Full correlation
Zhang et al., 2022b	sCLBP<br>Control	CSP<br>-	30<br>30	63%<br>57%	56.3 ± 9.7<br>55 ± 12.3	5.4 ± 2.4<br>-	3.7 ± 1	rs-fMRI	3 T<br>0.01 – 0.1 Hz	non-iso<br>(3.7×3.7×3)	AAL<br>(90)	B<br>(5 – 46)	GRETNA	Full correlation
Zhou et al., 2024	sCLBP – Group 1<br>sCLBP – Group 2<br>Control	CSP<br>CSP<br>-	28<br>31<br>28	54%<br>52%<br>50%	33.6 ± 7.3<br>34.2 ± 7.4<br>33.68 ± 7.4	NR<br>NR<br>-	6.6 ± 1.7<br>6.4 ± 1.7<br>-	rs-fMRI	3 T<br>0.01 – 0.1 Hz	iso<br>(3×3×3)	AAL<br>(90)	B<br>(5 – 27.5)	GRETNA	Full correlation

Note. AAL = Automatic anatomic labelling atlas. B = Binary. C-SCI = Complete spinal cord injury. CRPS = Chronic regional pain syndrome. CTN = Classic trigeminal neuralgia. CPP = Chronic primary pain. CSP = Chronic secondary pain. DKA = Desikan-Killiany Atlas. FCwAD = Functional constipation with anxiety/depression. FCwoAD = Functional constipation without anxiety/depression. IC-SCI = Incomplete spinal cord injury. IBS = Irritable bowel syndrome. NDPH = New daily persistent headache. NP = Neuropathic pain. NR = not reported. nsCLBP = Non-specific chronic low back pain. OA = Osteoarthritis. PHN = Postherpetic neuralgia. PNP = Polyneuropathic pain. rs-EEG = resting-state electroencephalogram. rs-fMRI = Resting-state functional magnetic resonance imaging. rs-MEG = resting-state magnetoencephalography. sCLBP = Specific chronic low back pain. W = Weighted. WAD = Whiplash-associated disorder.

Table 4.2: Study characteristics of studies investigating structural brain topology

	Population characteristics							Imaging characteristics				Network computation characteristics			
**Study**	**Group**	**Pain type (ICD-11)**	**Group size (n)**	**Sex (♂)**	**Age (years)**	**Pain duration (months)**	**Pain intensity (NRS)**	**Field strength (T)**	**Voxel dimensions (mm)**	**Gradient directions (n) + b-value (s/mm^2)**	**Model + (Tracto-graphy)**	**Parcellation (n)**	**Edge type + (Sparsity, %)**	**Soft-ware**	**Edge metric**
Chao et al., 2022	PDN<br>Control	CSP<br>-	24<br>24	63%<br>38%	60.1 ± 10.6<br>57.0 ± 9.9	34.8 ± 38.4<br>-	5.8 ± 2.1<br>-	3 T	non-iso<br>(3.1×3.1×3.3)	64<br>1500	DTI<br>(P)	HOA<br>(110)	B<br>(4.3 – 20)	BCT	SLC<br>(adj.)
Dai et al., 2021	Episodic migraine<br>Control	CPP<br>-	45<br>35	20%<br>37%	36.4 ± 13.7<br>41.5 ± 15.3	163.2 ± 124.1<br>-	6.4 ± 1.8<br>-	3 T	non-iso<br>(2.5×2.5×2.3)	34<br>1000	DTI<br>(NR)	AAL<br>(90)	W<br>(75)	GRETNA	SLC,<br>FA
Huang et al., 2021	CP/CPPS<br>Control	CSP<br>-	19<br>32	100%<br>100%	38.11 ± 9<br>33.9 ± 8.5	NR<br>-	NR<br>-	3 T	NR	30<br>1000	DTI<br>(D)	AAL<br>(90)	W<br>(none)	BCT	FA
Kurokawa et al., 2021	BMS<br>Control	CPP<br>-	14<br>11	0%<br>0%	57 ± 12.1<br>55.8 ± 8.1	71.3 ± 64.3<br>-	7.21 ± 2<br>-	3 T	iso<br>(2×2×2)	47; 46<br>1500; 3000	DTI<br>(P)	DKA<br>(84)	W<br>(none)	BCT	SLC
Lee et al., 2023	Episodic migraine<br>Control	CPP<br>-	59<br>30	14%<br>17%	38.4 ± 11.5<br>37.9 ± 8.7	128.5 ± 132.3<br>-	7.4 ± 1.6<br>-	3 T	non-iso<br>(2×2×2.3)	32<br>1000	DTI<br>(D)	AAL3<br>(166)	NR<br>(NR)	BRAPH	SLC
Li et al., 2017	Episodic migraine<br>Control	CPP<br>-	30<br>30	NR<br>NR	37.2 ± 6.8<br>36.4 ± 5	134.4 ± 79.2<br>-	5.5 ± 1.6<br>-	3 T	iso<br>(2×2×2)	60<br>1000	DTI<br>(P)	AAL<br>(90)	B<br>(NR)	NR	SLC
Liu et al., 2013	Episodic migraine<br>Control	CPP<br>-	26<br>26	0%<br>0%	34.6 ± 4.5<br>33.3 ± 3	141.6 ± 68.4<br>-	4.1 ± 0.8<br>-	3 T	non-iso<br>(1.8×1.8×3)	30<br>1000	DTI<br>(D)	HOA + Cereb<br>(300)	W<br>(10 – 20)	NR	FA
Liu et al., 2017	Episodic migraine<br>Control	CPP<br>-	71<br>50	NR<br>NR	22 ± 2.4<br>22 ± 2	56 ± 43.3<br>-	5.5 ± 1.7<br>-	3 T	iso<br>(2×2×2)	30<br>1000	DTI<br>(NR)	AAL<br>(90)	B<br>(none)	NR	FA
Matoso et al., 2024	Episodic migraine<br>Control	CPP<br>-	14<br>15	0%<br>0%	35.5 ± 8.7<br>30.8 ± 6.8	241.2 ± 132<br>-	6.6 ± 1.3<br>-	3 T	iso<br>(2×2×2)	32; 32; 60<br>400; 1000; 2000	DTI<br>(P)	AAL116-aggregated<br>(15)	NR<br>(NR)	BCT	SLC<br>(adj.)
Mei et al., 2024	Episodic migraine<br>Chronic migraine<br>Control	CPP<br>CPP<br>-	34<br>60<br>39	38%<br>27%<br>41%	36.8 ± 13.9<br>39.8 ± 14.2<br>35 ± 9.9	NR<br>NR<br>-	6.7 ± 1.3<br>7.2 ± 1.5<br>-	3 T	iso<br>(2×2×2)	50<br>1000	DTI<br>(D)	AAL<br>(90)	W<br>(NR)	GRETNA	FA
Pijnenburg et al., 2016	nsCLBP<br>Control	CPP<br>-	17<br>17	35%<br>29%	33.3 ± 7.9<br>31.8 ± 8.2	117.7 ± 98.4<br>-	4.5 ± 2<br>-	3 T	iso<br>(2.5×2.5×2.5)	60<br>1300	DTI<br>(D)	AAL<br>(116)	B<br>(1 – 9)	NR	SLC
Silvestro et al., 2021	Episodic migraine<br>Control	CPP<br>-	94<br>91	24%<br>36%	30.5 ± 8.1<br>30.7 ± 8.4	132 ± 92.4<br>-	8.1 ± 1<br>-	3 T	iso<br>(2.5×2.5×2.5)	32<br>1000	DTI<br>(P)	AAL<br>(90)	B<br>($p < 0.05$)	BCT	SLC<br>(adj.)
Tu et al., 2023	Fibromyalgia<br>Control	CPP<br>-	20<br>20	0%<br>0%	46.4 ± 12.4<br>42.1 ± 12.5	62.4 ± 61.2<br>-	7.2 ± 1.6<br>-	3 T	non-iso<br>(1.1×1.1×2)	60<br>1000	DTI<br>(P)	AAL<br>(90)	W<br>(10 – 34)	GRETNA	SLC<br>(adj.)
Wada et al., 2017	BMS<br>Control	CPP<br>-	14<br>14	0%<br>0%	50.9 ± NR<br>50.2 ± NR	NR<br>-	NR<br>-	1.5 T	iso<br>(2.5×2.5×2.5)	60<br>1000	DTI<br>(P)	DKA<br>(83)	W<br>(none)	BCT	SLC
Wu et al., 2020	PTNwoNC<br>PTNwNC<br>Control	CPP<br>CPP<br>-	22<br>23<br>45	36%<br>52%<br>49%	47.7 ± 9.2<br>53.3 ± 8.7<br>49.4 ± 11.6	59.6 ± 25.1<br>68.9 ± 40.2<br>-	4.5 ± 1.5<br>5.4 ± 2.1	3 T	non-iso<br>(2×2×2.2)	32<br>1000	DTI<br>(NR)	AAL<br>(90)	W<br>(NR)	NR	SLC
Yang et al., 2024	IC-SCI with NP<br>C-SCI with NP<br>Control	CSP<br>CSP<br>-	15<br>20<br>32	60%<br>75%<br>81%	42.5 ± 18.1<br>39.3 ± 16.3<br>44.1 ± 12.2	85 ± 109.1<br>87.5 ± 84.1<br>-	5.4 ± 3.7<br>6.6 ± 3.5<br>-	3 T	iso<br>(2×2×2)	64<br>1000	DTI<br>(D)	AAL<br>(90)	NR<br>(none)	GRETNA	FA
Zhang et al., 2022a	CTN<br>Control	CSP<br>-	20<br>34	45%<br>35%	54.6 ±10.8<br>55 ± 6.8	49.4 ± 39<br>-	7.9 ± 0.9<br>-	3 T	non-iso<br>(2×2×3)	64<br>1000	DTI<br>(D)	Brainnetome<br>(246)	B<br>(absolute:<br>3 SLCs)	GRETNA	SLC<br>(adj.)

Note. AAL = Automatic anatomic labelling atlas. B = Binary. BMS = Burning mouth syndrome. C-SCI = Complete spinal cord injury. CP/CPPS = Chronic prostatitis/chronic pelvic pain syndrome. CPP = Chronic primary pain. CSP = Chronic secondary pain. CTN = Classic trigeminal neuralgia. D = Deterministic. DKA = Desikan-Killiany Atlas. FA = Fractional anisotropy. HOA = Harward-Oxford Atlas. IC-SCI = Incomplete spinal cord injury. NP = Neuropathic pain. NR = Not reported. NRS = Numeric rating scale. nsCLBP = Non-specific chronic low back pain. P = Probabilistic. PDN = Painful diabetic neuropathy. PTNwNC = Primary trigeminal neuralgia with neurovascular compression. PTNwoNC = Primary trigeminal neuralgia without neurovascular compression. SLC = Streamline count. W = Weighted.

Table 4.3: Assessment of psychological comorbidity in structural topology studies

Study	Assessment of psychological comorbidity		
	Population criteria	PROM	Not assessed
Chao et al., 2022			x
Dai et al., 2021	x		
Huang et al., 2021	x	CP	
Kurokawa et al., 2021		CP	
Lee et al., 2023			x
Li et al., 2017			x
Liu et al., 2013			x
Liu et al., 2017	x	CP	
Matoso et al., 2024	x		
Mei et al., 2024		CP	
Pijnenburg et al., 2016			x
Silvestro et al., 2021		CP	
Tu et al., 2023		CP & HC	
Wada et al., 2017			x
Wu et al., 2020			x
Yang et al., 2024	x		
Zhang et al., 2022a	x	CP & HC	

Note. PROM = Patient reported outcome measure. CP = Chronic pain. HC = Healthy control.

Table 4.4: Assessment of psychological comorbidity in functional topology studies

Study	Assessment of psychological comorbidity		
	Population criteria	PROM	Not reported
Balenzuela et al., 2010			x
Barroso et al., 2021		CP	
De Pauw et al., 2020	x		
Duan et al., 2021	x	CP & HC	
Fauchon et al., 2021	x		
Huang et al., 2019	x	CP & HC	
Kang et al., 2024			x
Kaplan et al., 2019		CP & HC	
Larkin et al., 2021	x	CP	
Lin et al., 2022	x	CP	
Liu et al., 2011			x
Liu et al., 2012			x
Liu et al., 2018	x		
Mansour et al., 2016		CP	
Mao et al., 2024		CP & HC	
Nieboer et al., 2020			x
Qiu et al., 2023	x	CP	
Shi et al., 2019			x
Shi et al., 2020			x
Ta Dinh et al., 2019	x	CP	
Tu et al., 2019	x	CP	
Wang et al., 2022	x	CP & HC	
Wang et al., 2024	x		
Wu et al., 2016			x
Yang et al., 2023	x	CP & HC	
Yang et al., 2024	x		
Yang et al., 2025	x	CP	
Zhang et al., 2014	x		
Zhang et al., 2017	x		
Zhang et al., 2022a	x	CP & HC	
Zhang et al., 2022b	x		
Zhou et al., 2024	x		
Note. PROM = Patient reported outcome measure. CP = Chronic pain. HC = Healthy control.			

5 Appendix 5: Results not included in meta-analyses

Table 5.1: Study results excluded from meta-analyses due to insufficient data or insufficient reporting (marked by asterisk)

	Integration				Segregation				Centrality			Resilience	Other
	Lp	*Diameter*	*Density*	*Eglob*	*Cp*	*Transitivity*	*Q*	*N modules*	*Avg. BC*	*Avg. within-module degree*	*PC*	*Assortativity*	*Small-worldness*
**FUNCTIONAL (fMRI)**													
Barroso et al., 2021*							⇔		⇔				⇔
Huang et al., 2019									↓		⇔		
Larkin et al., 2021								⇔					
Fauchon et al., 2021										↑			
Balenzuela et al., 2010*								⇔					
Lin et al., 2022*	⇔			⇔	⇔								
Shi et al., 2020*			⇔			⇔						⇔	
**STRUCTURAL (dMRI)**													
Chao et al., 2022									↓				
Lee et al., 2023		↓										⇔	

Note. * = excluded from meta-analysis due to incomplete reporting. ↓ = Decreased in chronic pain. ↑ = Increase in chronic pain. ⇔ No difference between chronic pain and healthy controls. Lp = Characteristic path length. Eglob = Global efficiency. Cp = Clustering coefficient. Q = Modularity. N modules = Number of modules. Avg. = Average. BC = Betweenness centrality. PC = Participation coefficient.

Table 5.2: Study results excluded from meta-analyses due to imaging modality

	Integration				Segregation		Other		
	Lp	*Diameter*	*Eglob*	*Avg. Degree*	*Cp*	*Eloc*	*Small-wordlness*	*Leaf fraction*	*Tree hierarchy*
**FUNCTIONAL (EEG & MEG)**									
Qiu et al., 2023	⇔		↑ (1-4 Hz; else ⇔)			⇔			
Ta Dinh et al., 2019			↓ (60 – 100 Hz; else ⇔)		⇔		⇔		
Wu et al., 2016	↑ (4 – 8; 80 - 250 Hz; else ⇔)			↑ (0.1-1 Hz; else ⇔)	↑ (4 – 8 Hz; else ⇔)				
Nieboer et al., 2020		⇔						⇔	⇔

Note. ↓ = Decreased in chronic pain. ↑ = Increase in chronic pain. ⇔ No difference between chronic pain and healthy controls. Lp = Characteristic path length. Eglob = Global efficiency. Cp = Clustering coefficient. Eloc = Local efficiency.

6 Appendix 6 – Results of functional brain topology (FT) meta-analysis

6.1 Forest plots of FT – Overall

Figure 6.1 Forest plot of FT – Overall – Clustering coefficient

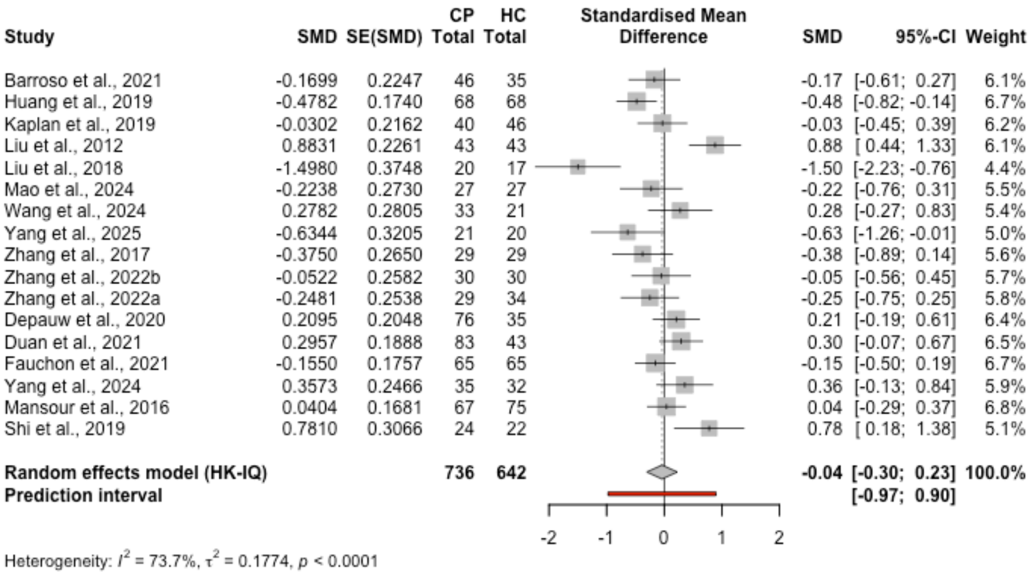

Figure 6.2 Forest plot of FT – Overall – Global efficiency

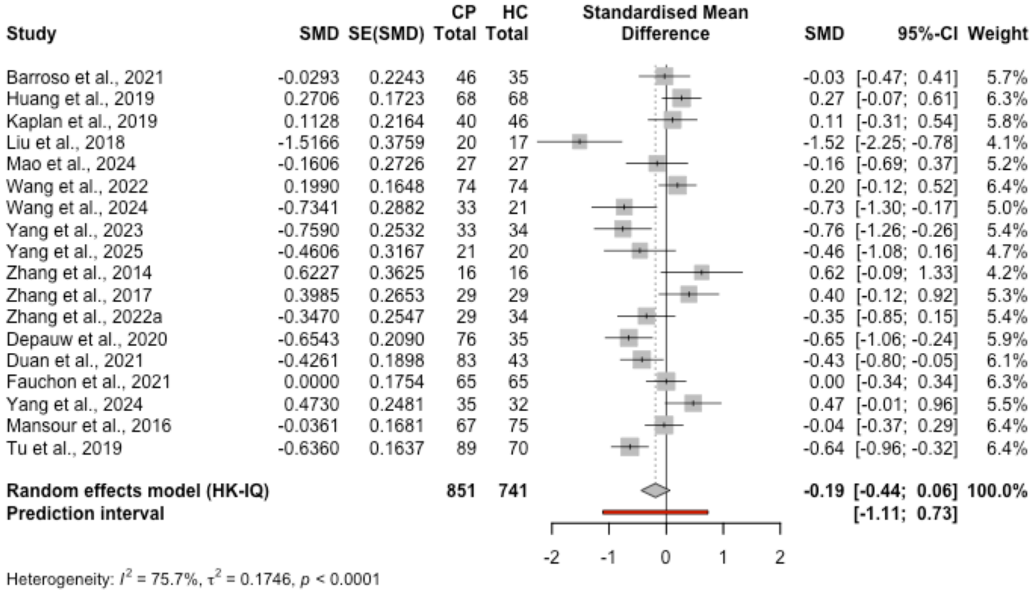

Figure 6.3 Forest plot of FT – Overall – Local efficiency

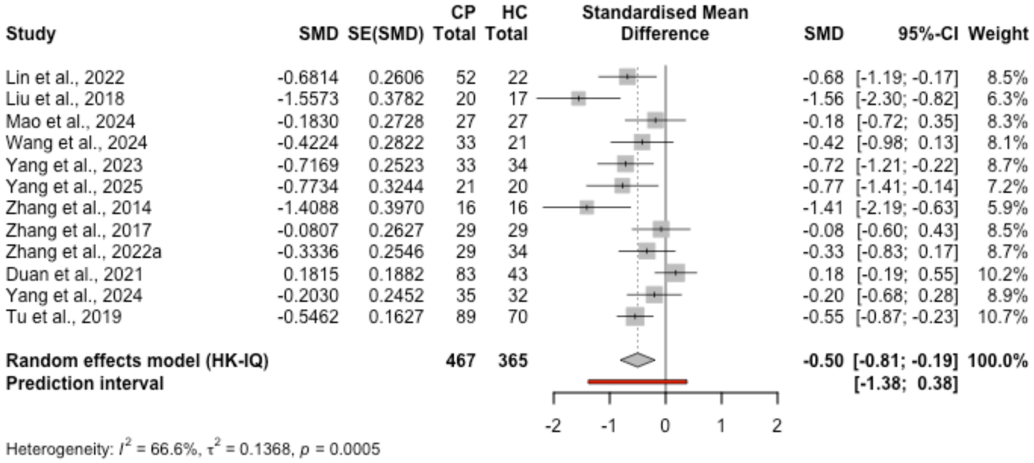

Figure 6.4 Forest plot of FT – Overall – Normalised clustering coefficient

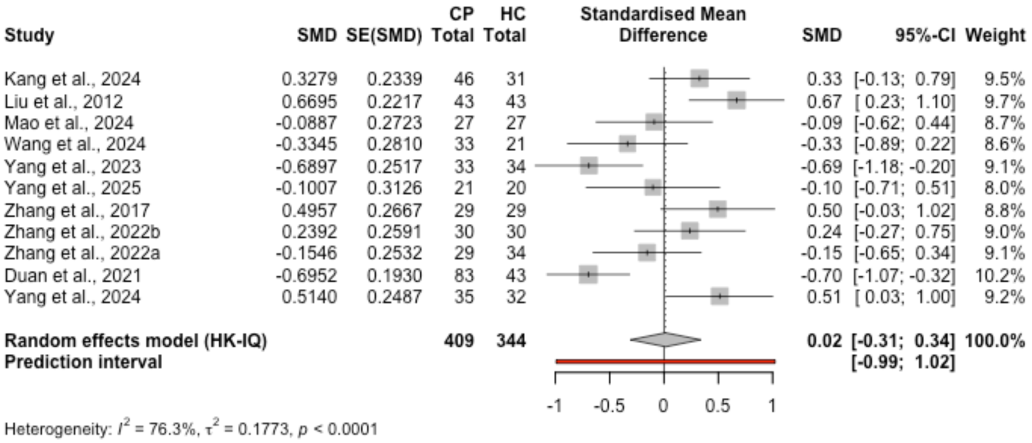

Figure 6.5 Forest plot of FT – Overall – Normalised characteristic path length

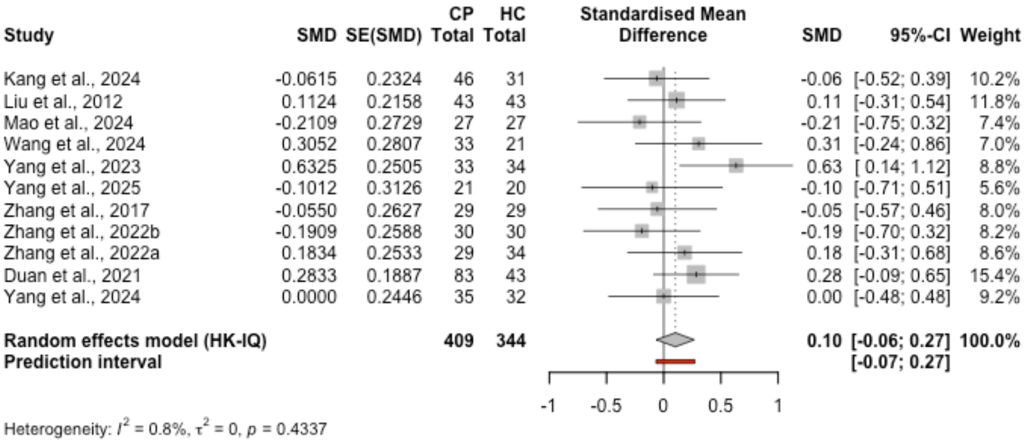

Figure 6.6 Forest plot of FT – Overall – Characteristic path length

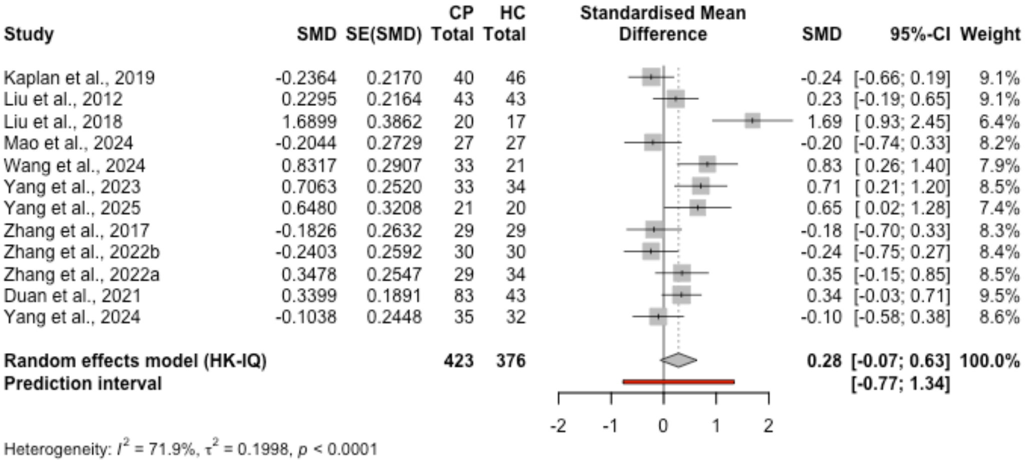

Figure 6.7 Forest plot of FT – Overall – Normalised mutual information

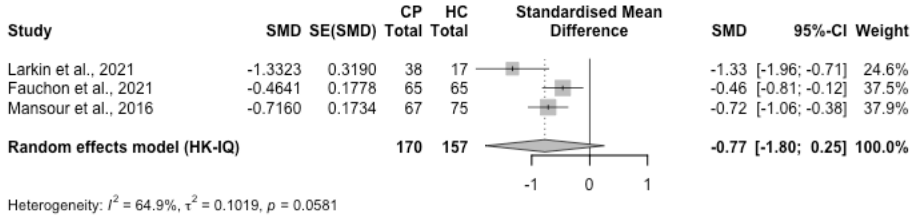

Figure 6.8 Forest plot of FT – Overall – Modularity

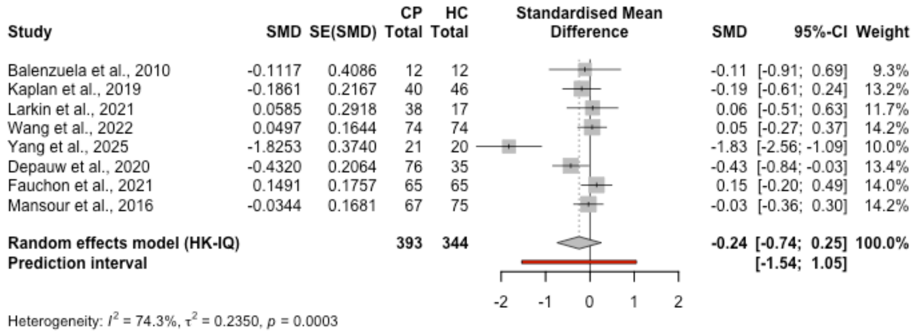

Figure 6.9 Forest plot of FT – Overall – Small-worldness

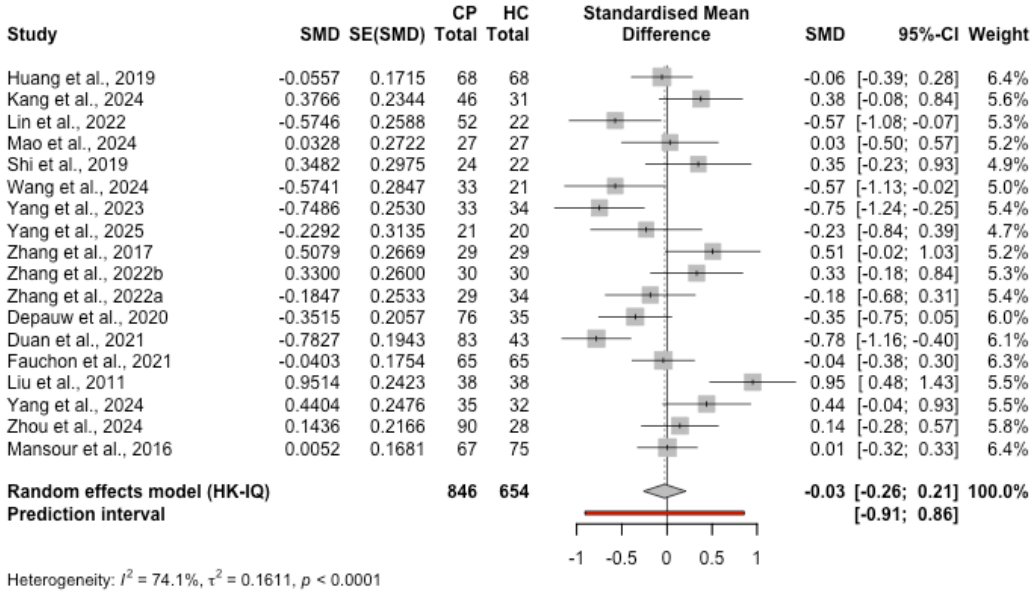

6.2 Forest plots of FT subgroup analysis: Chronic primary pain (CPP)

Figure 6.10 Forest plot of FT – Subgroup CPP – Clustering coefficient

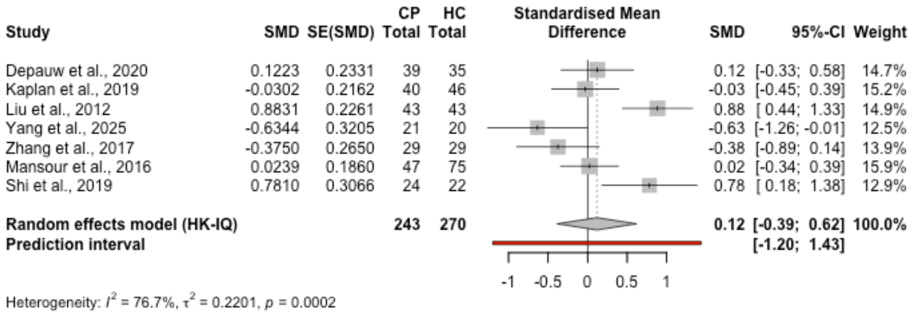

Figure 6.11 Forest plot of FT – Subgroup CPP – Global efficiency

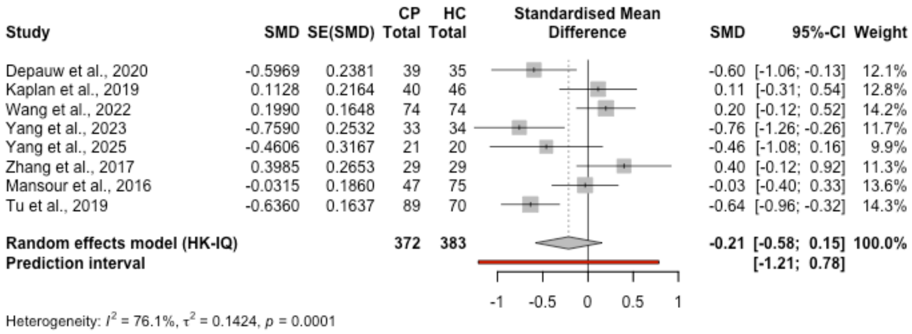

Figure 6.12 Forest plot of FT – Subgroup CPP – Local efficiency

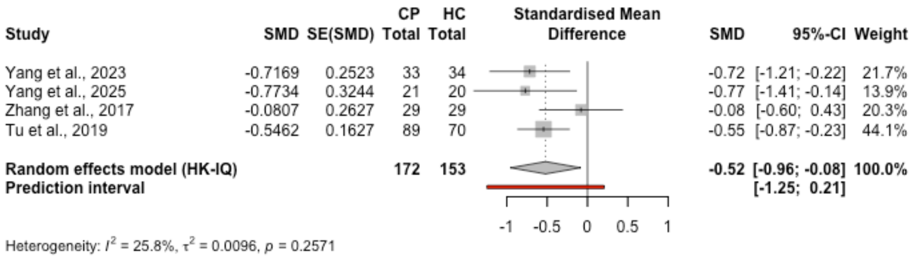

Figure 6.13 Forest plot of FT – Subgroup CPP – Normalised clustering coefficient

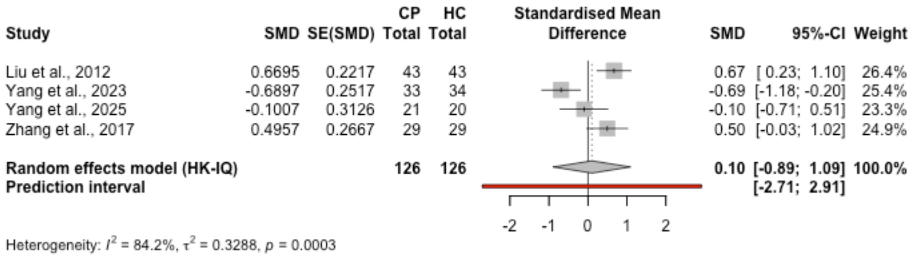

Figure 6.14 Forest plot of FT – Subgroup CPP – Normalised characteristic path length

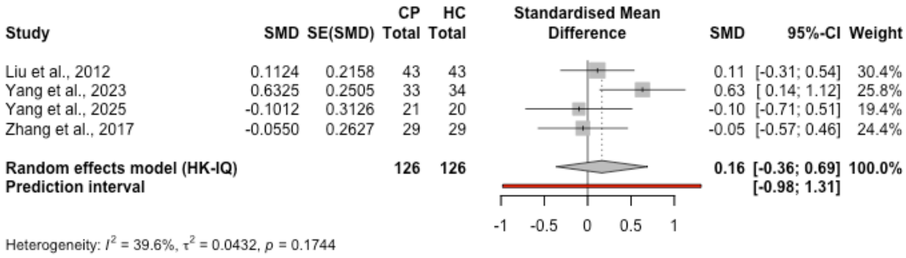

Figure 6.15 Forest plot of FT – Subgroup CPP – Characteristic path length

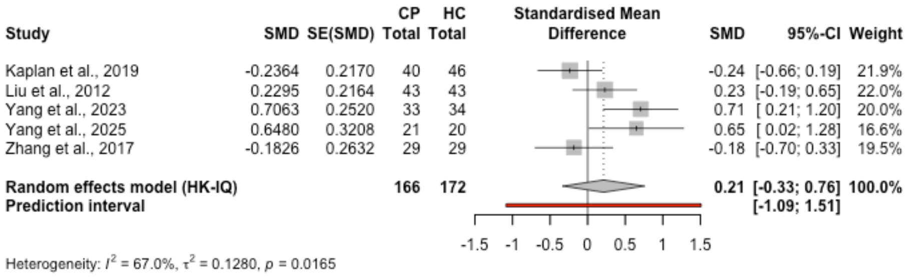

Figure 6.16 Forest plot of FT – Subgroup CPP – Modularity

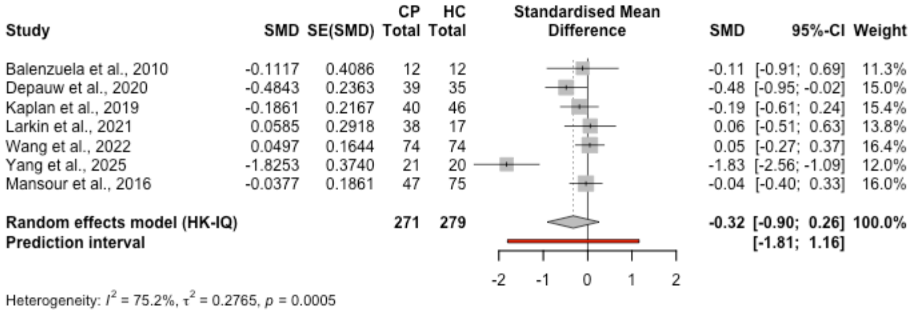

Figure 6.17 Forest plot of FT – Subgroup CPP – Small-worldness

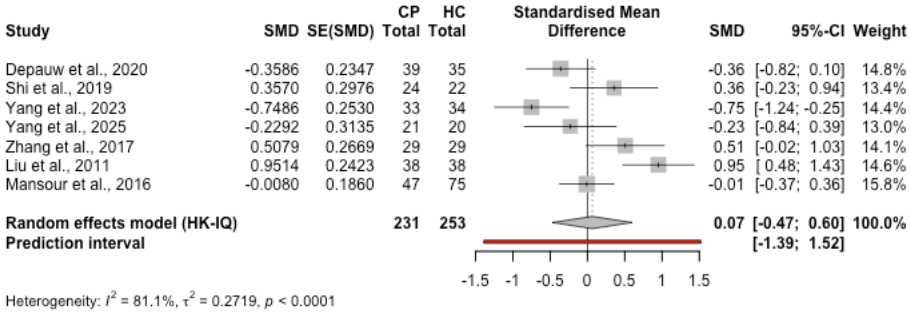

6.3 Forest plots of FT subgroup analysis: Chronic secondary pain (CSP)

Figure 6.18 Forest plot of FT – Subgroup CSP – Clustering coefficient

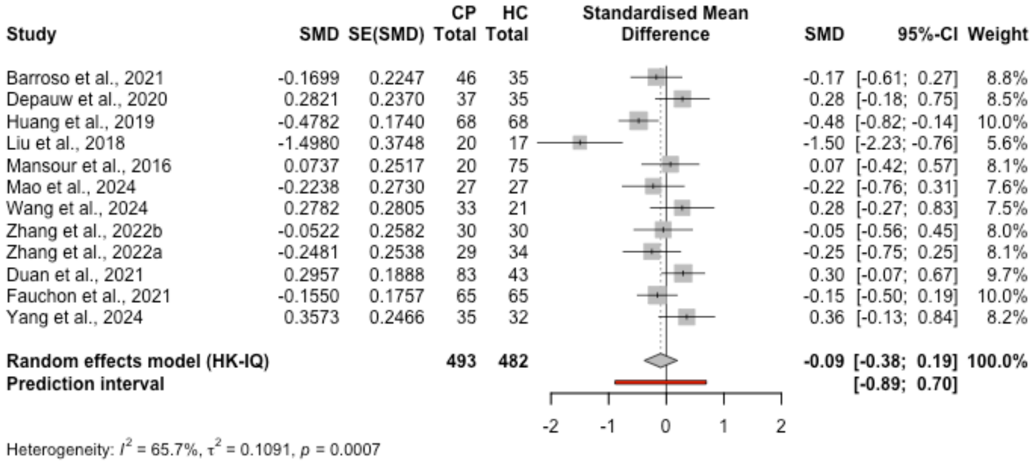

Figure 6.19 Forest plot of FT – Subgroup CSP – Global efficiency

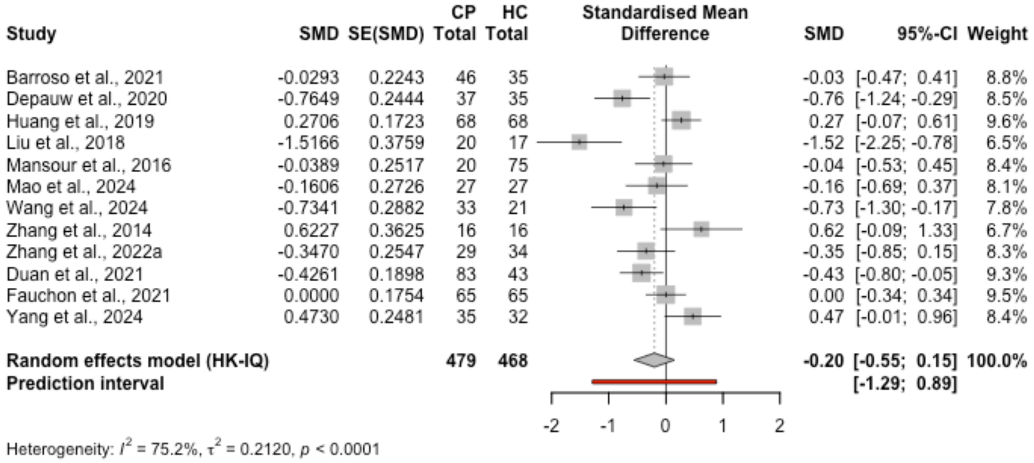

Figure 6.20 Forest plot of FT – Subgroup CSP – Local efficiency

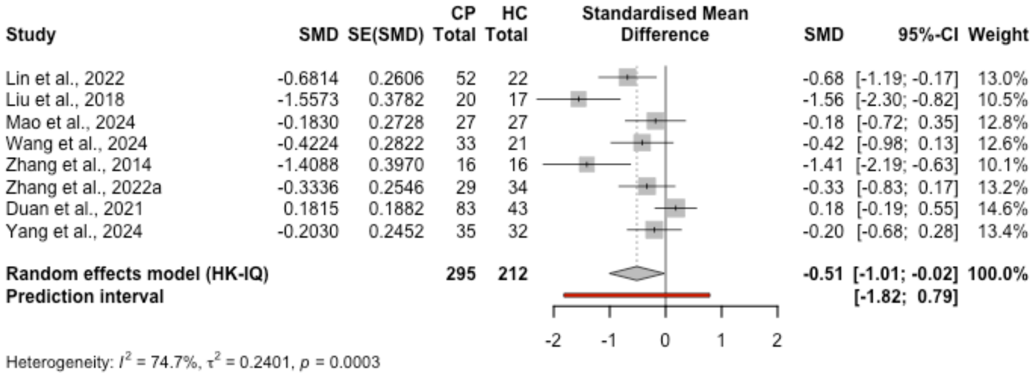

Figure 6.21 Forest plot of FT – Subgroup CSP – Normalised clustering coefficient

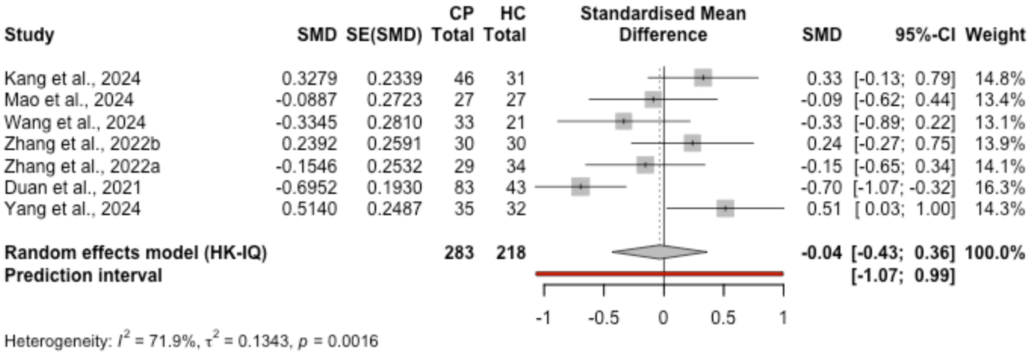

Figure 6.22 Forest plot of FT – Subgroup CSP – Normalised characteristic path length

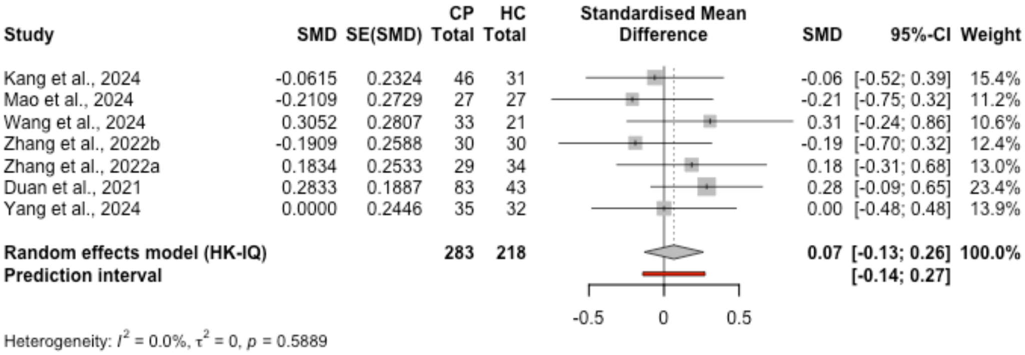

Figure 6.23 Forest plot of FT – Subgroup CSP – Characteristic path length

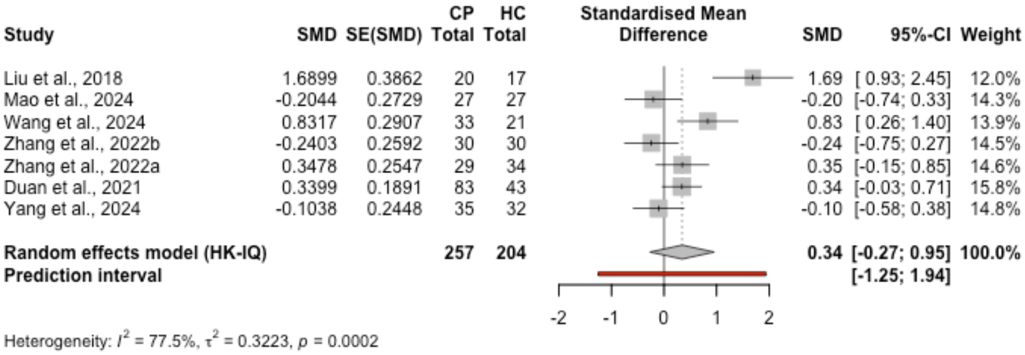

Figure 6.24 Forest plot of FT – Subgroup CSP – Modularity

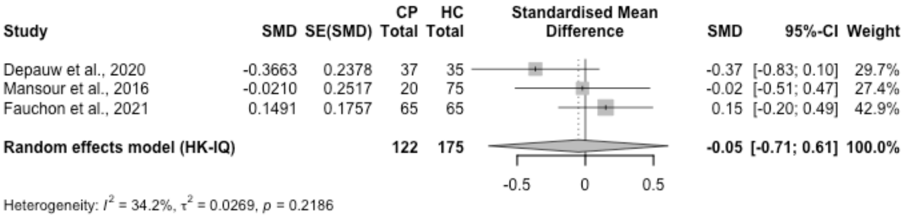

Figure 6.25 Forest plot of FT – Subgroup CSP – Small-worldness

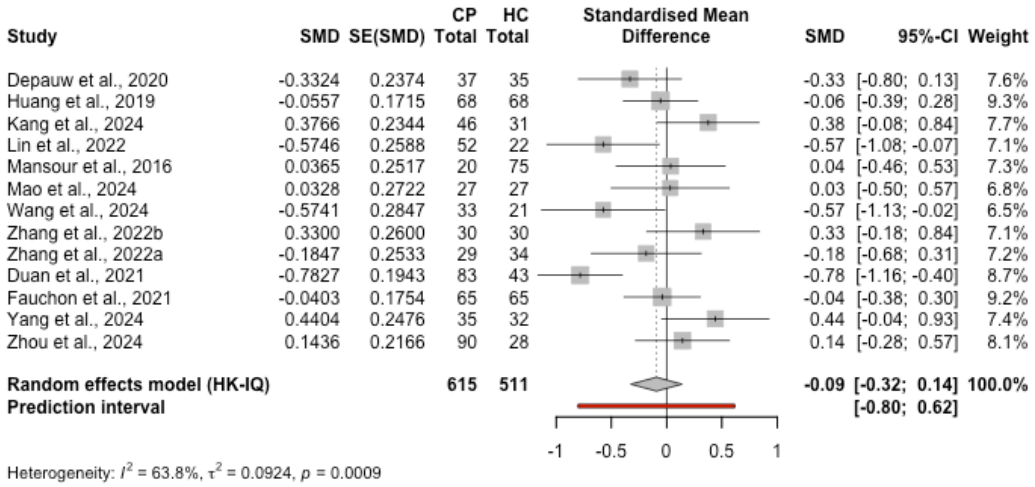

6.4 Sensitivity analysis of functional topology meta-analysis

Table 6.1 Sensitivity analysis for functional topology: Removal of outliers

Analysis	Original estimate: SMD [95%-CI]	Sensitivity estimate: SMD [95%-CI]	Change from initial analysis
Functional topology – Overall			
<u>Cp – outlier removal:</u><br>Liu et al., 2012<br>Liu et al., 2018	-0.04<br>[−0.30; 0.23]	-0.03<br>[-0.21; 0.15]	Marginal change.
<u>Eglob – outlier removal:</u><br>Liu et al., 2018	-0.19<br>[−0.44; 0.06]	-0.13<br>[-0.35; 0.09]	Marginal change.
<u>Eloc – outlier removal:</u><br>Liu et al., 2018<br>Duan et al., 2021	**-0.50<br>[−0.81; -0.19]**	**-0.48<br>[-0.68; -0.29]**	Lower CI moved from large to moderate value. Upper CI moved from very small to small value.
<u>Lp – outlier removal:</u><br>Liu et al., 2018	0.28<br>[−0.07; 0.63]	0.18<br>[-0.08; 0.44]	SMD reduced. CI narrowed.
<u>Q – outlier removal:</u><br>Yang et al., 2025	-0.24<br>[−0.74; 0.25]	-0.05<br>[-0.25; 0.14]	SMD moved from small to very small value. CI narrowed.
<u>Gamma – outlier removal:</u><br>Duan et al., 2021	-0.02<br>[−0.31; 0.34]	0.10<br>[-0.21; 0.41]	SMD moved from very small negative to very small positive value. CI narrowed.
<u>Small-worldness – outlier removal:</u><br>Duan et al., 2021<br>Liu et al., 2011	-0.03<br>[−0.26; 0.21]	-0.03<br>[-0.23; 0.16]	Marginal change.
Functional topology – Subgroup analysis: CPP			
<u>Q – outlier removal:</u><br>Yang et al., 2025	-0.32<br>[−0.90; 0.26]	-0.10<br>[-0.33; 0.14]	SMD moved from small to very small value. CI narrowed.
Functional topology – Subgroup analysis: CSP			
<u>Cp – outlier removal:</u><br>Liu et al., 2018	-0.09<br>[−0.38; 0.19]	-0.02<br>[-0.22; 0.18]	Marginal change.
<u>Eglob – outlier removal:</u><br>Liu et al., 2018	-0.20<br>[−0.55; 0.15]	-0.11<br>[-0.40; 0.18]	Marginal change.
<u>Small-worldness – outlier removal:</u><br>Duan et al., 2021	-0.09<br>[−0.32; 0.14]	-0.02<br>[-0.22; 0.17]	Marginal change.

Table 6.2 Sensitivity analysis for functional topology: Removal of studies with SMD estimation based on p-value

Analysis	Original estimate: SMD [95%-CI]	Sensitivity estimate: SMD [95%-CI]	Change from initial analysis
Functional topology – Overall			
Cp – removal: Shi et al., 2019	-0.04 [−0.30; 0.23]	-0.08 [-0.34; 0.18]	Marginal change.
Eglob – removal: Tu et al., 2019	-0.19 [−0.44; 0.06]	-0.15 [-0.42; 0.10]	Marginal change.
Eloc – removal: Tu et al., 2019	**-0.50 [−0.81; -0.19]**	**-0.50 [-0.85; -0.16]**	Marginal change.
Functional topology – Subgroup analysis: CPP			
Cp – removal: Shi et al., 2019	0.12 [−0.39; 0.62]	0.02 [-0.51; 0.56]	SMD reduced. Lower CI moved from small to moderate value.
Eglob – removal: Tu et al., 2019	-0.21 [−0.58; 0.15]	-0.14 [-0.54; 0.26]	Marginal change.
Eloc – removal: Tu et al., 2019	**-0.52 [−0.96; −0.08]**	-0.51 [-1.47; 0.45]	Upper CI moved from very small negative to small positive value. CI encloses zero.

7 Appendix 7 – Results of structural brain topology (ST) meta-analysis

7.1 Forest plots of ST – Overall

Figure 7.1 Forest plot of ST – Overall – Clustering coefficient

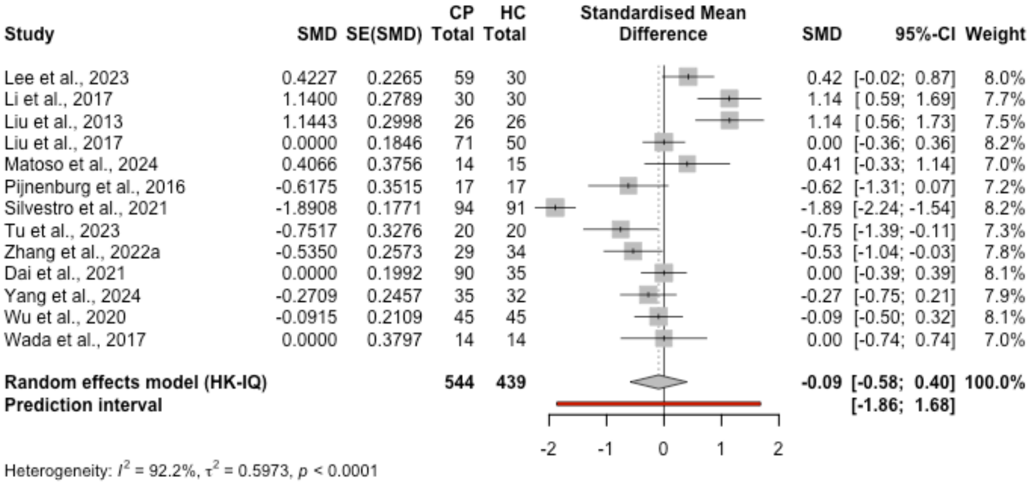

Figure 7.2 Forest plot of ST – Overall – Global efficiency

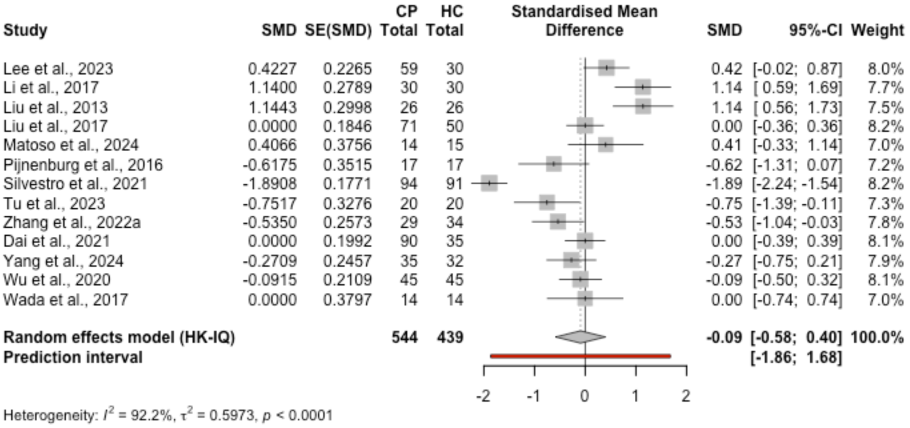

Figure 7.3 Forest plot of ST – Overall – Local efficiency

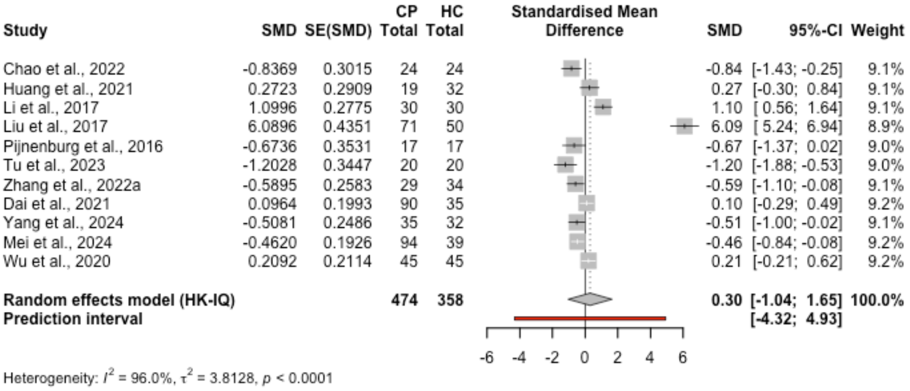

Figure 7.4 Forest plot of ST – Overall – Normalised clustering coefficient

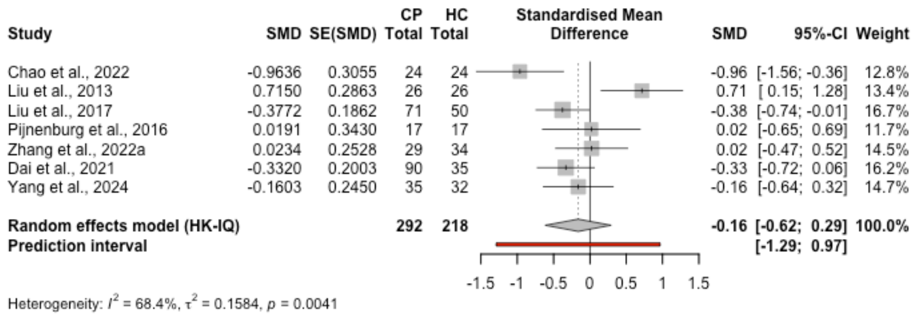

Figure 7.5 Forest plot of ST – Overall – Normalised characteristic path length

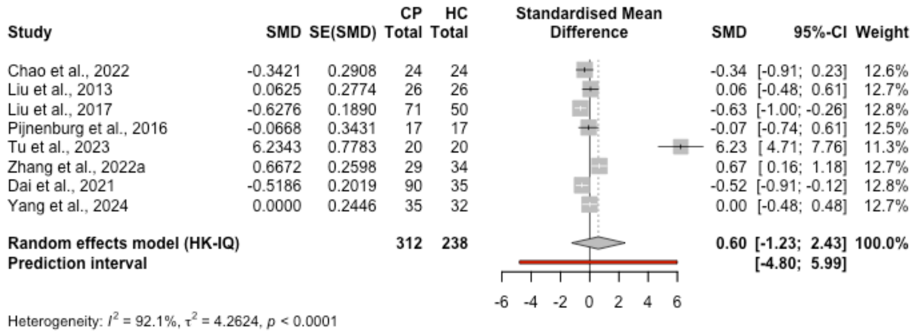

Figure 7.6 Forest plot of ST – Overall – Characteristic path length

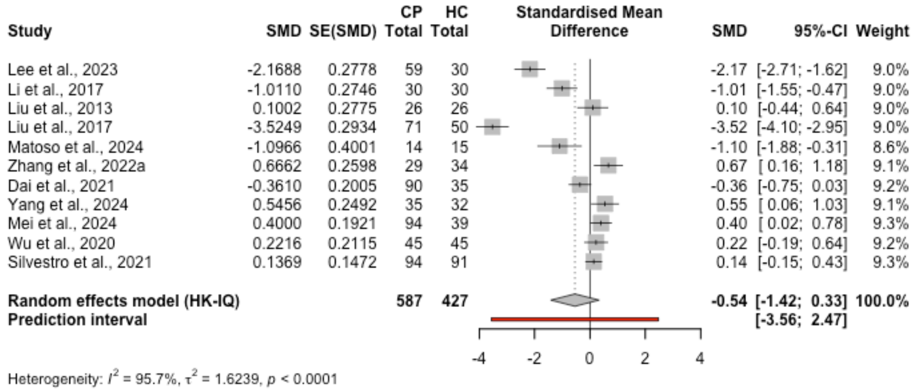

Figure 7.7 Forest plot of ST – Overall – Small-worldness

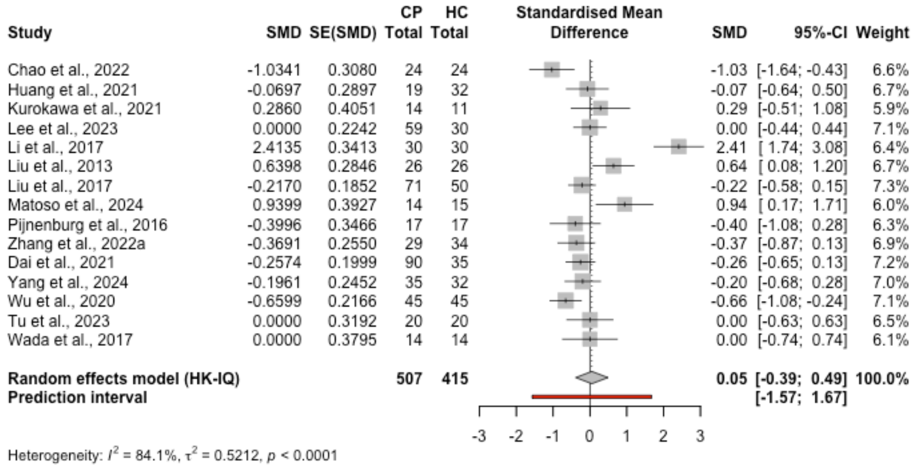

7.2 Forest plots of ST subgroup analysis: Chronic primary pain (CPP)

Figure 7.8 Forest plot of ST – Subgroup CPP – Average degree

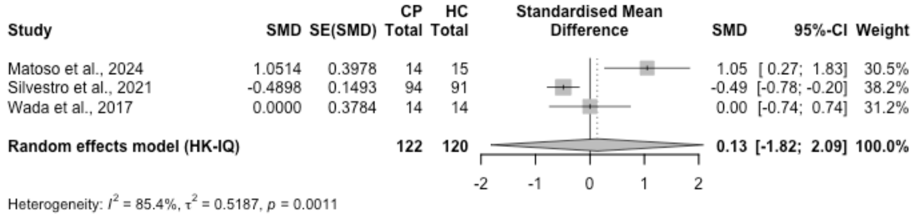

Figure 7.9 Forest plot of ST – Subgroup CPP – Clustering coefficient

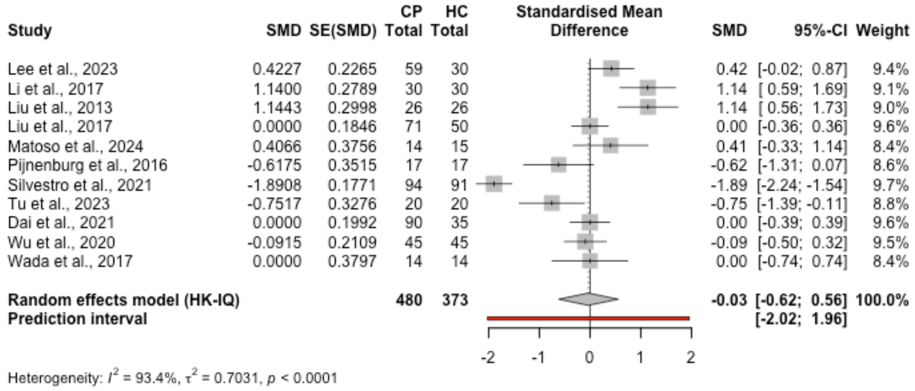

Figure 7.10 Forest plot of ST – Subgroup CPP – Global efficiency

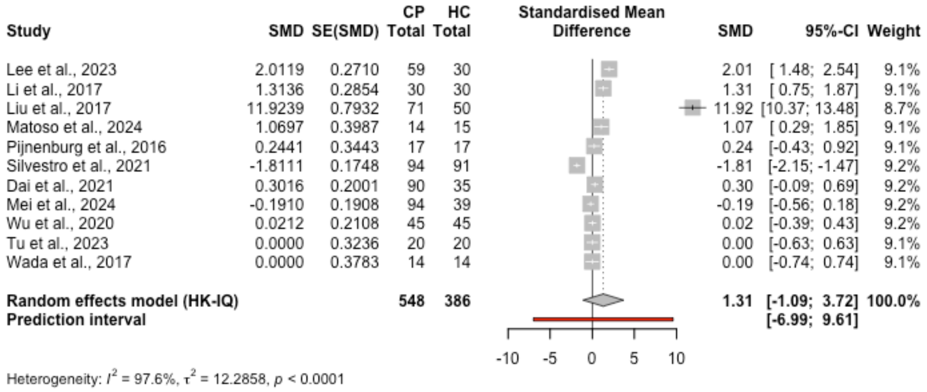

Figure 7.11 Forest plot of ST – Subgroup CPP – Local efficiency

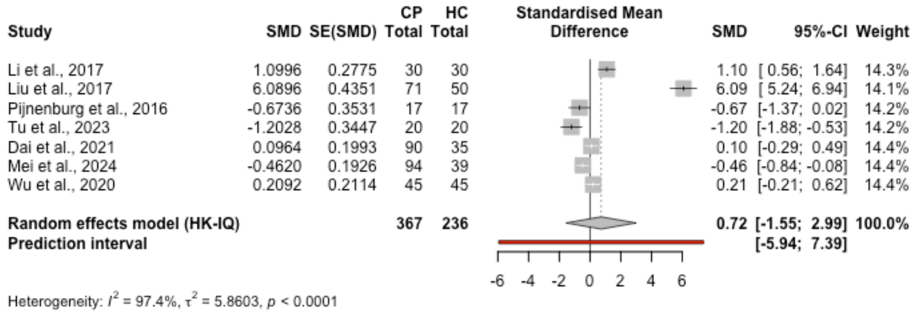

Figure 7.12 Forest plot of ST – Subgroup CPP – Normalised clustering coefficient

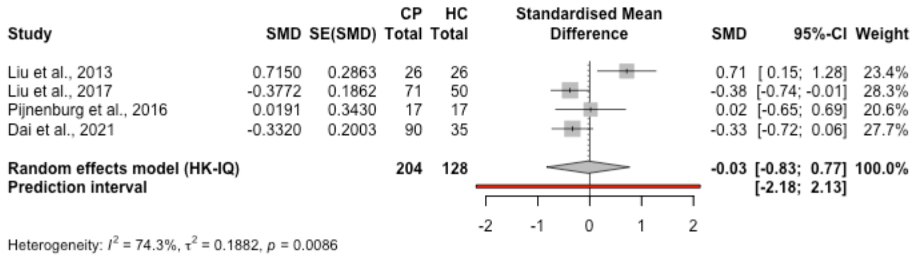

Figure 7.13 Forest plot of ST – Subgroup CPP – Normalised characteristic path length

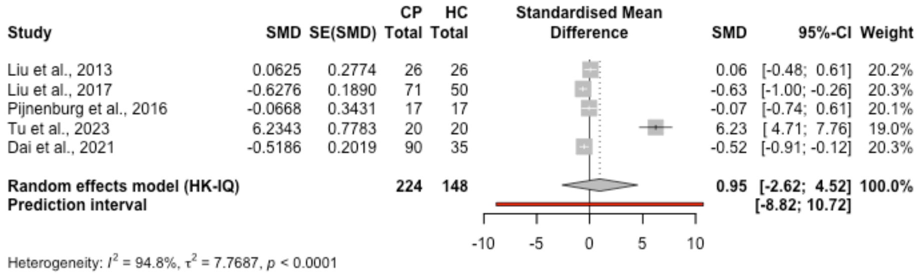

Figure 7.14 Forest plot of ST – Subgroup CPP – Characteristic path length

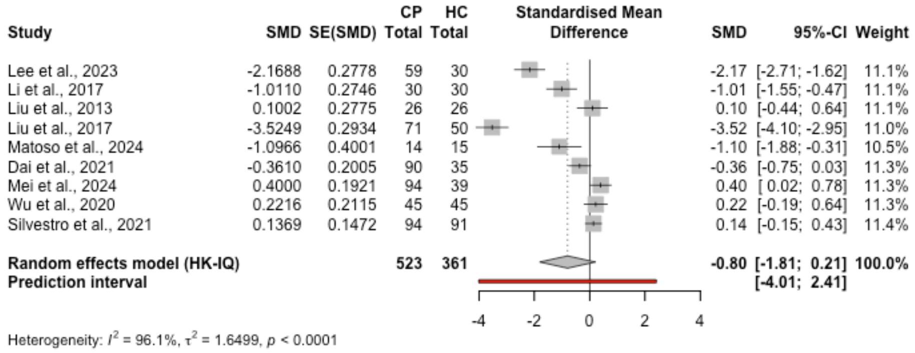

Figure 7.15 Forest plot of ST – Subgroup CPP – Modularity

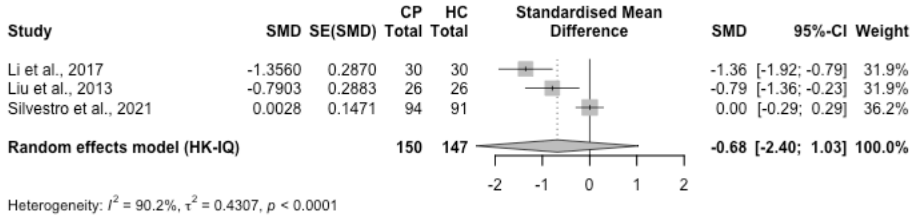

Figure 7.16 Forest plot of ST – Subgroup CPP – Small-worldness

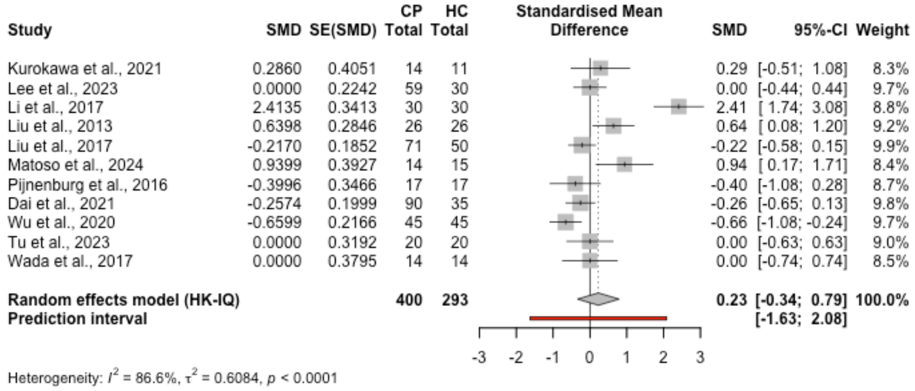

7.3 Forest plots of ST subgroup analysis: Chronic secondary pain (CSP)

Figure 7.17 Forest plot of ST – Subgroup CSP – Global efficiency

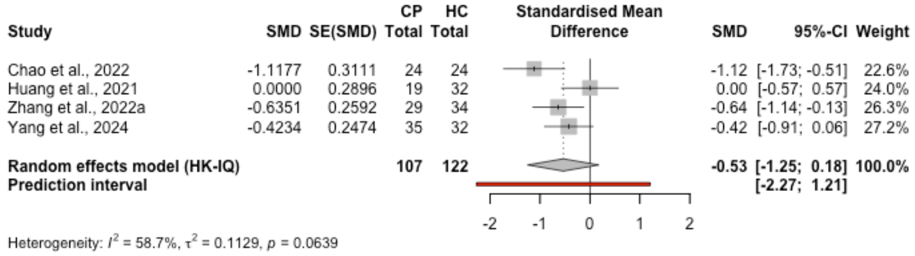

Figure 7.18 Forest plot of ST – Subgroup CSP – Local efficiency

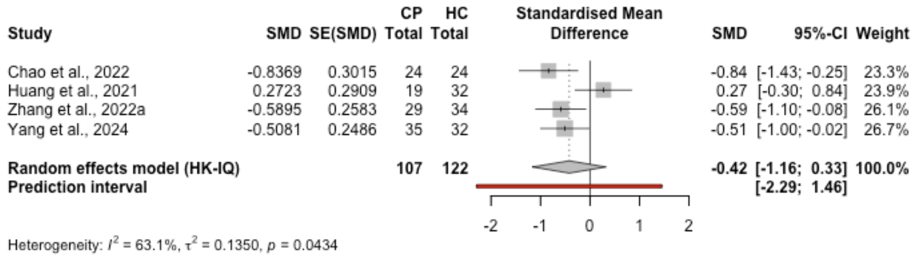

Figure 7.19 Forest plot of ST – Subgroup CSP – Normalised clustering coefficient

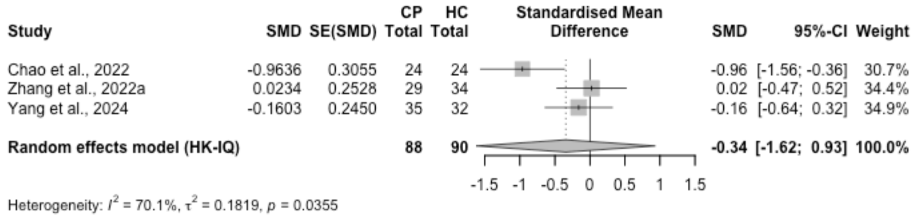

Figure 7.20 Forest plot of ST – Subgroup CSP – Normalised characteristic path length

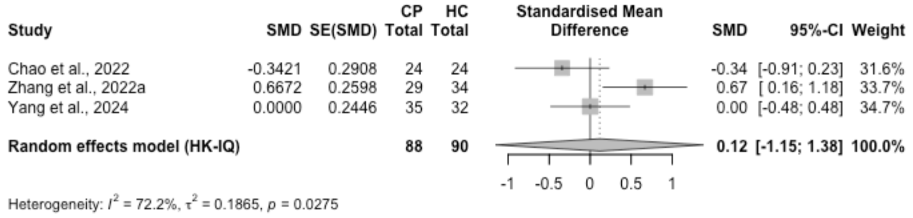

Figure 7.21 Forest plot of ST – Subgroup CSP – Small-worldness

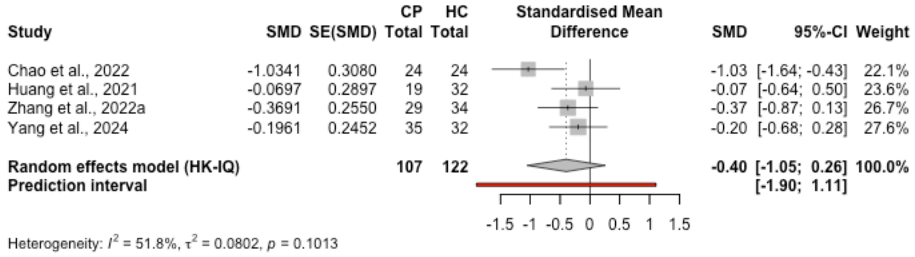

7.4 Sensitivity analysis of structural topology meta-analysis

Table 7.1 Sensitivity analysis for structural topology: Removal of outliers

Analysis	Original estimate: SMD [95%-CI]	Sensitivity estimate: SMD [95%-CI]	Change from initial analysis
Structural topology – Overall			
Cp – outlier removal:<br>Li et al., 2017<br>Liu et al., 2013<br>Silvestro et al., 2021	-0.09<br>[−0.58; 0.40]	-0.12<br>[-0.38; 0.14]	CI narrowed.
Eglob – outlier removal:<br>Liu et al., 2017<br>Silvestro et al., 2021	0.81<br>[−0.93; 2.54]	0.19<br>[-0.31; 0.70]	SMD moved from large to very small value. CI narrowed.
Eloc – outlier removal:<br>Liu et al., 2017	0.30<br>[−1.04; 1.65]	-0.24<br>[-0.71; 0.23]	SMD moved from positive low to negative low value. CI narrowed.
Lp – outlier removal:<br>Lee et al., 2023<br>Liu et al., 2017	-0.54<br>[−1.42; 0.33]	0.01<br>[-0.47; 0.45]	SMD moved from negative moderate to very low positive value. Lower CI moved from large to moderate value. Upper CI increased.
Lambda – outlier removal:<br>Tu et al., 2023	0.60<br>[−1.23; 2.43]	-0.14<br>[-0.56; 0.28]	SMD moved from positive moderate to very small negative value. CI narrowed.
Small-worldness – outlier removal:<br>Chao et al., 2022<br>Li et al., 2017	0.05<br>[−0.39; 0.49]	-0.08<br>[-0.32; 0.16]	SMD moved from positive to negative very small value. CI narrowed.
Structural topology – Subgroup analysis: CPP			
Cp – outlier removal:<br>Li et al., 2017<br>Silvestro et al., 2021	-0.03<br>[−0.62; 0.56]	0.07<br>[-0.35; 0.48]	SMD moved from negative to positive very small value. CI narrowed.
Eglob – outlier removal:<br>Liu et al., 2017<br>Silvestro et al., 2021	1.31<br>[−1.09; 3.72]	0.52<br>[-0.07; 1.11]	SMD moved from large to moderate value. CI narrowed.
Eloc – outlier removal:<br>Liu et al., 2017	0.72<br>[−1.55; 2.99]	-0.14<br>[-0.95; 0.68]	SMD moved from positive large to negative very small value. CI narrowed.
Lp – outlier removal:<br>Liu et al., 2017	-0.80<br>[−1.81; 0.21]	-0.45<br>[-1.19; 0.29]	SMD moved from large to small value. CI narrowed.
Lambda – outlier removal:<br>Tu et al., 2023	0.95<br>[−2.62; 4.52]	-0.35<br>[-0.88; 0.18]	SMD moved from positive large to negative small value. CI narrowed.
Small-worldness – outlier removal:<br>Li et al., 2017	0.23<br>[−0.34; 0.79]	-0.02<br>[-0.35; 0.31]	SMD moved from positive small to negative very small value. CI narrowed.
Structural topology – Subgroup analysis: CSP			
No outliers detected			

Table 7.2 Sensitivity analysis for structural topology: Removal of studies with SMD estimation based on p-value

Analysis	Original estimate: SMD [95%-CI]	Sensitivity estimate: SMD [95%-CI]	Change from initial analysis
Structural topology – Main analysis			
Cp – removal:<br>Wada et al., 2017	-0.09<br>[−0.58; 0.40]	-0.10<br>[-0.63; 0.44]	Marginal change.
Eglob – removal:<br>Tu et al., 2023	0.81<br>[−0.93; 2.54]	0.94<br>[-1.10; 2.98]	Marginal change.

Wada et al., 2017			
Lp – removal:<br>Silvestro et al., 2021	-0.54<br>[−1.42; 0.33]	-0.61<br>[-1.58; 0.36]	Marginal change.
Small-wordness – removal:<br>Tu et al., 2023<br>Wada et al., 2017	0.05<br>[−0.39; 0.49]	0.06<br>[-0.45; 0.58]	Marginal change.
Structural topology – Subgroup analysis: CPP			
Average degree – removal:<br>Silvestro et al., 2021<br>Wada et al., 2017	0.13<br>[−1.82; 2.09]	only 1 study<br>(Matoso et al., 2024)	No sufficient data for meta-analysis
Cp – removal:<br>Wada et al., 2017	-0.03<br>[−0.62; 0.55]	-0.10<br>[-0.63; 0.44]	Marginal change.
Eglob – removal:<br>Tu et al., 2023<br>Wada et al., 2017	1.31<br>[−1.09; 3.72]	0.94<br>[-1.10; 2.98]	SMD reduced but stayed at large value.
Lp – removal:<br>Silvestro et al., 2021	-0.79<br>[−1.80; 0.22]	-0.61<br>[-1.58; 0.36]	SMD moved from large to moderate value.
Small-worldness – removal:<br>Tu et al., 2023<br>Wada et al., 2017	0.22<br>[−0.34; 0.79]	0.06<br>[-0.45; 0.58]	SMD moved from small to very small value.
Structural topology – Subgroup analysis: CSP			
No outliers detected			

8 PRISMA Checklist

Section and Topic	Item #	Checklist item	Location where item is reported
**TITLE**			
Title	1	Identify the report as a systematic review.	Title
**ABSTRACT**			
Abstract	2	See the PRISMA 2020 for Abstracts checklist.	Abstract
**INTRODUCTION**			
Rationale	3	Describe the rationale for the review in the context of existing knowledge.	Introduction
Objectives	4	Provide an explicit statement of the objective(s) or question(s) the review addresses.	Introduction
**METHODS**			
Eligibility criteria	5	Specify the inclusion and exclusion criteria for the review and how studies were grouped for the syntheses.	/Eligibility criteria
Information sources	6	Specify all databases, registers, websites, organisations, reference lists and other sources searched or consulted to identify studies. Specify the date when each source was last searched or consulted.	/Search strategy
Search strategy	7	Present the full search strategies for all databases, registers and websites, including any filters and limits used.	/Search strategy + Appendix 1
Selection process	8	Specify the methods used to decide whether a study met the inclusion criteria of the review, including how many reviewers screened each record and each report retrieved, whether they worked independently, and if applicable, details of automation tools used in the process.	/Screening
Data collection process	9	Specify the methods used to collect data from reports, including how many reviewers collected data from each report, whether they worked independently, any processes for obtaining or confirming data from study investigators, and if applicable, details of automation tools used in the process.	/Data extraction
Data items	10a	List and define all outcomes for which data were sought. Specify whether all results that were compatible with each outcome domain in each study were sought (e.g. for all measures, time points, analyses), and if not, the methods used to decide which results to collect.	/Data extraction
	10b	List and define all other variables for which data were sought (e.g. participant and intervention characteristics, funding sources). Describe any assumptions made about any missing or unclear information.	/Data extraction
Study risk of bias assessment	11	Specify the methods used to assess risk of bias in the included studies, including details of the tool(s) used, how many reviewers assessed each study and whether they worked independently, and if applicable, details of automation tools used in the process.	/Risk of bias and meta-analysis quality assessment
Effect measures	12	Specify for each outcome the effect measure(s) (e.g. risk ratio, mean difference) used in the synthesis or presentation of results.	/Statistical analysis
Synthesis methods	13a	Describe the processes used to decide which studies were eligible for each synthesis (e.g. tabulating the study intervention characteristics and comparing against the planned groups for each synthesis (item #5)).	/Statistical analysis
	13b	Describe any methods required to prepare the data for presentation or synthesis, such as handling of missing summary statistics, or data conversions.	/Statistical analysis + /Results/Data handling

Section and Topic	Item #	Checklist item	Location where item is reported
	13c	Describe any methods used to tabulate or visually display results of individual studies and syntheses.	/Statistical analysis
	13d	Describe any methods used to synthesize results and provide a rationale for the choice(s). If meta-analysis was performed, describe the model(s), method(s) to identify the presence and extent of statistical heterogeneity, and software package(s) used.	/Statistical analysis
	13e	Describe any methods used to explore possible causes of heterogeneity among study results (e.g. subgroup analysis, meta-regression).	/Statistical analysis
	13f	Describe any sensitivity analyses conducted to assess robustness of the synthesized results.	/Statistical analysis
Reporting bias assessment	14	Describe any methods used to assess risk of bias due to missing results in a synthesis (arising from reporting biases).	/Statistical analysis
Certainty assessment	15	Describe any methods used to assess certainty (or confidence) in the body of evidence for an outcome.	Risk of bias and meta-analysis quality assessment
**RESULTS**			
Study selection	16a	Describe the results of the search and selection process, from the number of records identified in the search to the number of studies included in the review, ideally using a flow diagram.	Fig. 1
	16b	Cite studies that might appear to meet the inclusion criteria, but which were excluded, and explain why they were excluded.	/Study selection and characteristics
Study characteristics	17	Cite each included study and present its characteristics.	/Study selection and characteristics; Tbl. 1, Tbl.2
Risk of bias in studies	18	Present assessments of risk of bias for each included study.	Fig. 2
Results of individual studies	19	For all outcomes, present, for each study: (a) summary statistics for each group (where appropriate) and (b) an effect estimate and its precision (e.g. confidence/credible interval), ideally using structured tables or plots.	Appx. 5; Appx. 6
Results of syntheses	20a	For each synthesis, briefly summarise the characteristics and risk of bias among contributing studies.	/Risk of bias
	20b	Present results of all statistical syntheses conducted. If meta-analysis was done, present for each the summary estimate and its precision (e.g. confidence/credible interval) and measures of statistical heterogeneity. If comparing groups, describe the direction of the effect.	Fig. 3; Fig. 4; Appx. 5; Appx. 6
	20c	Present results of all investigations of possible causes of heterogeneity among study results.	Meta-analysis/Functional topology + Structural topology
	20d	Present results of all sensitivity analyses conducted to assess the robustness of the synthesized results.	/Meta-analysis/Functional topology + Structural topology
Reporting biases	21	Present assessments of risk of bias due to missing results (arising from reporting biases) for each synthesis assessed.	/Meta-analysis/Data handling
Certainty of evidence	22	Present assessments of certainty (or confidence) in the body of evidence for each outcome assessed.	Fig. 3; Fig. 4
**DISCUSSION**			

Section and Topic	Item #	Checklist item	Location where item is reported
Discussion	23a	Provide a general interpretation of the results in the context of other evidence.	/Discussion
	23b	Discuss any limitations of the evidence included in the review.	/Discussion/Heterogeneity, …
	23c	Discuss any limitations of the review processes used.	/Discussion/Limitations
	23d	Discuss implications of the results for practice, policy, and future research.	/Discussion
**OTHER INFORMATION**			
Registration and protocol	24a	Provide registration information for the review, including register name and registration number, or state that the review was not registered.	Methods
	24b	Indicate where the review protocol can be accessed, or state that a protocol was not prepared.	Methods
	24c	Describe and explain any amendments to information provided at registration or in the protocol.	Methods/Deviations from study protocol
Support	25	Describe sources of financial or non-financial support for the review, and the role of the funders or sponsors in the review.	Acknowledgement
Competing interests	26	Declare any competing interests of review authors.	Conflict of interest
Availability of data, code and other materials	27	Report which of the following are publicly available and where they can be found: template data collection forms; data extracted from included studies; data used for all analyses; analytic code; any other materials used in the review.	Code and data availability

From: Page MJ, McKenzie JE, Bossuyt PM, Boutron I, Hoffmann TC, Mulrow CD, et al. The PRISMA 2020 statement: an updated guideline for reporting systematic reviews. BMJ 2021;372:n71. doi: 10.1136/bmj.n71
